# Comprehensive map of age-associated splicing changes across human tissues and their contributions to age-associated diseases

**DOI:** 10.1038/s41598-018-29086-2

**Published:** 2018-07-19

**Authors:** Kun Wang, Di Wu, Haoyue Zhang, Avinash Das, Mahashweta Basu, Justin Malin, Kan Cao, Sridhar Hannenhalli

**Affiliations:** 1 Center for Bioinformatics and Computational Biology, University of Maryland, College Park, MD 20742 USA; 2 Department of Cell Biology and Molecular Genetics, University of Maryland, College Park, MD 20742 USA; 3 Computational Biology Branch, National Center for Biotechnology Information, National Institutes of Health, Bethesda, MD 20892 USA

## Abstract

Alternative splicing contributes to phenotypic diversity at multiple biological scales, and its dysregulation is implicated in both ageing and age-associated diseases in human. Cross-tissue variability in splicing further complicates its links to age-associated phenotypes and elucidating these links requires a comprehensive map of age-associated splicing changes across multiple tissues. Here, we generate such a map by analyzing ~8500 RNA-seq samples across 48 tissues in 544 individuals. Employing a stringent model controlling for multiple confounders, we identify 49,869 tissue-specific age-associated splicing events of 7 distinct types. We find that genome-wide splicing profile is a better predictor of biological age than the gene and transcript expression profiles, and furthermore, age-associated splicing provides additional independent contribution to age-associated complex diseases. We show that the age-associated splicing changes may be explained, in part, by concomitant age-associated changes of the upstream splicing factors. Finally, we show that our splicing-based model of age can successfully predict the relative ages of cells in 8 of the 10 paired longitudinal data as well as in 2 sets of cell passage data. Our study presents the first systematic investigation of age-associated splicing changes across tissues, and further strengthening the links between age-associated splicing and age-associated diseases.

## Introduction

Almost all multi-exon genes in human exhibit alternative splicing^1,2^, which alongside transcriptional regulation, significantly contribute to the transcriptomic as well as phenotypic diversity at multiple biological scales^3^. Much like transcription, splicing is highly regulated, by both genetic and environmental factors, and its dysregulation is implicated in, among other things, normal ageing as well as age-associated diseases^4–7^.

Normal ageing is associated with systemic changes in cellular processes involving both transcriptional and post-transcriptional controls^8^. While some of the changes in molecular processes are caused by age-related changes in the cellular environment, it is possible that molecular changes may further contribute to the ageing process, and to age-related diseases such as hypertension and cardiovascular diseases. Moreover, such age-associated changes in the transcriptional and post-transcriptional regulation are likely to vary across tissues and organs. While age-associated gene expression changes across several tissues have been previously reported^9,10^, similar investigations of age-associated splicing changes are limited.

Mazin *et al*. have previously reported age-associated splicing changes in two brain regions^11^, and Tollervey *et al*.^12^ have investigated age-associated splicing and transcript expression across normal and Alzheimer’s disease samples. However, these few previous studies: (1) focused only on a single or very few tissues in contrast to 48 primary tissues included in our study, (2) investigated only exon skipping events while we have studied 7 types of splicing events (exon skipping, alternative 5’, alternative 3’, mutually exclusive exon, alternative first exon, alternative last exon, and intron retention), (3) are based on very few individuals (around 35), in contrast to 177 individuals on average per tissue in our study, and highly importantly, (4) in contrast to our study, do not explicitly control for batch effect and potential hidden confounding factors, which may lead to false positives. Our study addresses these limitations in the previous studies toward a comprehensive investigation of age-associated splicing changes across human tissues, which may provide insights into age-related diseases mediated by splicing changes.

Based on ~8500 RNA-seq samples from 544 donors across 48 tissues in the Genotype-Tissue Expression dataset (GTEx version 6)^13^, here we report a comprehensive detection of age-associated splicing changes across tissues in human. Using a stringent model, we identified 49,869 age-associated splicing events of 7 distinct types^14^, including 17,447 exon-skipping events, across the 48 tissues.

We found that age-associated splicing changes are prevalent in all tissues and although the specific events are largely tissue-specific, overall the corresponding genes are involved in biological processes linked with ageing, such as mitochondrial function, DNA repair, DNA damage, apoptosis^15^, etc. Interestingly, in the majority of tissues the tissue-specific splicing profile of an individual is more predictive of their biological age than their gene and isoform expression levels. Likewise, in modeling age-related complex diseases, notably hypertension, the age-associated splicing events provide significant information in addition to gene expression profile, age, and gender. We show that age-associated splicing events can partly be explained by a concomitant change in the expression of their upstream splicing factors, thus elucidating a potential mechanism underlying age-associated splicing changes. Finally, we show that our splicing-based model of age can successfully predict the relative age of cells in 10 paired longitudinal data derived from the same individuals over time, as well as in fibroblast cell lines across multiple passages.

Overall, we report the first systematic genome-wide analysis of age-associated splicing events spanning 7 types of splicing events across 48 primary tissues, paving the way for future investigations of links between alternative splicing and ageing, and age-related diseases.

## Results

### Age-associated splicing events are prevalent in most tissues and are largely tissue-specific

**Figure 1:**
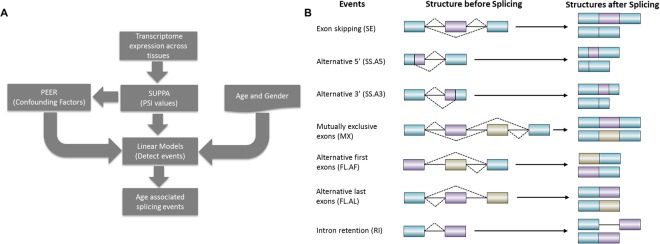
(**A**) Overall pipeline to detect age-associated splicing events. (**B**) Seven types of alternative splicing events before and after splicing. Blue rectangles represent constitutive portion of the exons. Purple and beige rectangles represent the alternatively used portion of the exons. Solid lines represent introns. Dashed lines connect the ends of alternatively spliced out portions of the gene. The splicing event structures before splicing and the generated isoform structures after splicing are shown on left and right side respectively.

Our overall pipeline is illustrated in Fig. 1A, and the details are provided in the Methods section. Briefly, we obtained a total of ~8500 expression samples across 48 tissues (the ones having at least 50 donor samples) and a total of 544 donors from GTEx^13^. The number of samples for each tissue and distributions of age and gender are shown in Fig. S1. Based on GENCODE annotations^16^, we compiled 163,505 alternative splicing events of 7 different types (Fig. 1B), and estimated sample-specific PSI values (Percent Splicing Index) for each event in each sample. A linear regression model was used to identify age-associated splicing events. To ensure sufficient statistical power, we only analyzed 48 tissues with at least 50 samples. Moreover, in a particular tissue, we only analyzed the events that could be quantified in at least 50 samples. More specifically, in a given tissue, we only analyzed the genes that are expressed in at least 50 individuals in the tissue. We thus analyzed a total of 163,505 splicing events spanning 7 types of events across the 48 tissues; a total of 3,723,596 tests; We describe the number of tested events across 7 types of events and 48 tissues in Supplementary Table S5.

**Figure 2:**
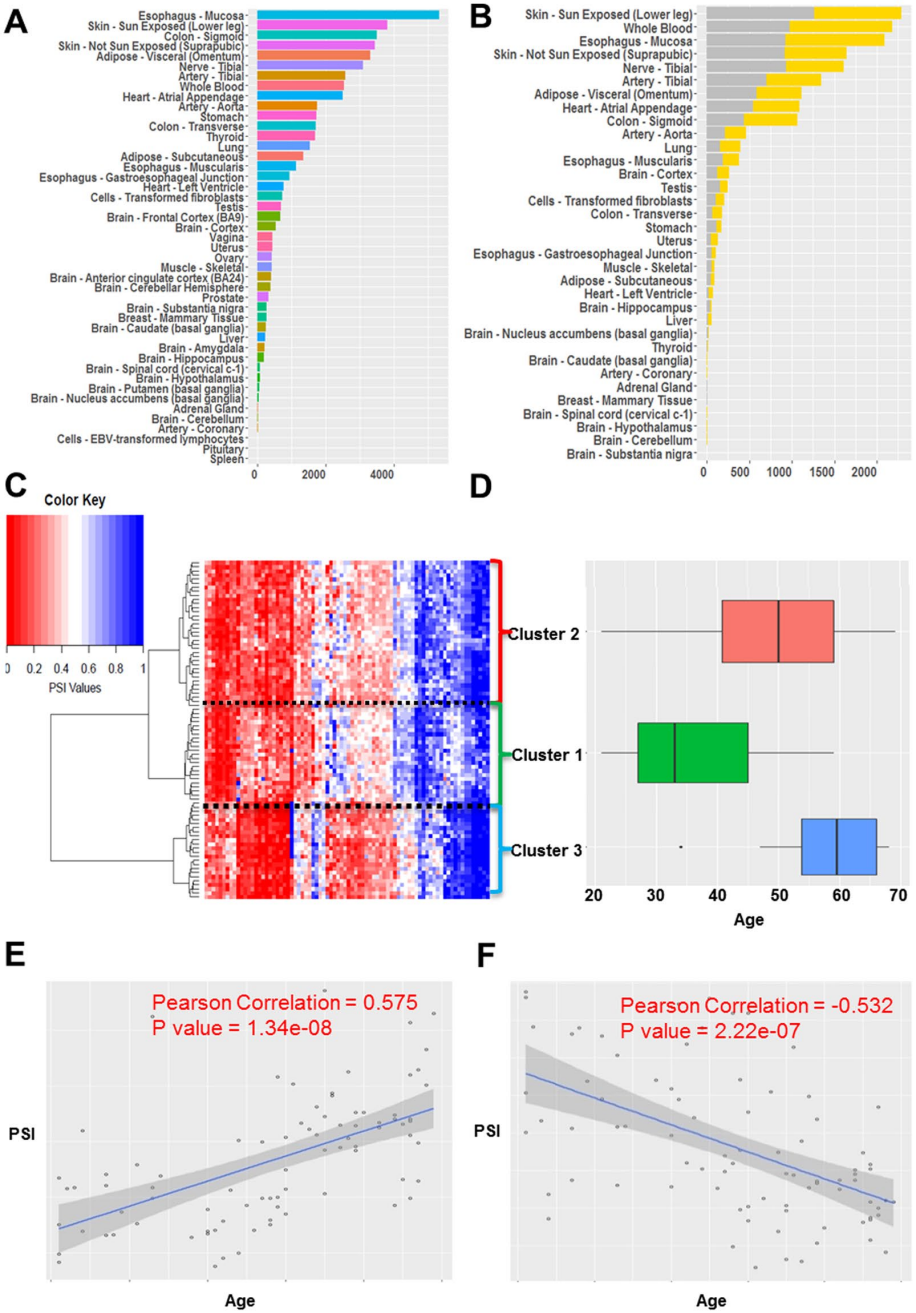
Summary of significant age-associated splicing events. (**A**) Number of significant age-associated splicing events across 48 tissues. (**B**) Number of significant up-regulated (increased with aging) and down-regulated (decreased with aging) exon skipping events across 48 tissues in gray and yellow color respectively. (**C**) Hierarchical biclustering of top age-associated exon skipping events across individuals, based on PSI value of each event; the columns represent events and the rows represent 83 individuals with age information; blue indicates higher PSI and red indicates lower PSI. (**D**) Box plot of age distributions of the three identified clusters of individuals. (**E**) Scatter plot illustrating an up-regulated cassette exon event (COL6A3: chr2:238285987–238287279:238287878–238289558) in Uterus. (**F**) Scatter plot illustrating a down-regulated cassette exon event (NFE2L1:chr17:46133960–46134394:46134483–46134706) in Uterus.

Summarized in Fig. 2A, overall 49,869 events (1.3%) were found to be significantly associated with ageing (FDR < = 0.05 and permutation p-value < = 0.05; see Methods) in at least one tissue; on average 1,018 events were detected in each tissue. We ascertained that the number of significant events detected in a tissue is not correlated with sample size (Supplementary Note 1). In addition, we show that our results are robust to the potential confounding by human ancestry (detailed in Supplementary Note 2). Figure 2B specifically summarizes the exon skipping event. In Supplementary Table S1 and Supplementary Fig. S2 we provide a detailed summary of significant age-associated events across 48 tissues for each type of splicing event. Age-associated splicing changes are found to be most abundant in Skin (Sun exposed) and Esophagus-Mucosa; interestingly, both these tissues are composed of epithelial cells and are most exposed to external environment, have a well-established effect of ageing^17^.

To further illustrate age-associated splicing events, Fig. 2C shows clustering of samples using the sample-specific PSI values of significant age-associated exon skipping in uterus, revealing subgroups with distinct age distributions (Fig. 2D; cluster 2> cluster 1: Wilcoxon p-value = 3.7e-04; cluster 3> cluster 2: p-value = 7.8e-3). While inter-cluster differences in age distribution are expected, interestingly, the three splicing-based clusters reflect three important reproductive/hormonal stages in females^18^: the median age of individuals in cluster 1 is 33 years which roughly corresponds to age of first child birth, and the median age of individuals in cluster 2 is 50 years which roughly corresponds to the onset of menopause, while the individuals in cluster 3 are 60 years old on average corresponding to post-menopause. Figure 2E,F illustrate two examples of significantly age-associated events in the uterus. Figure 2E shows an exon skipping event in COL6A3, a procollagen gene important in the extracellular matrix organization, previously shown to be linked to ageing in rat muscle tissue^19^. In addition, COL6A3 is related to different stages of pregnancy in mouse uterus tissue^20^. Figure 2F shows an exon skipping event in a nuclear factor gene NFE2L1, whose worm ortholog SKN-1 has been linked to lifespan extension^21^. In addition SKN-1 is significantly related to collagen expression^22^ which is critical for uterus. Interestingly, these two genes are also age-dependent in the other 6 and 7 tissues respectively.

**Figure 3:**
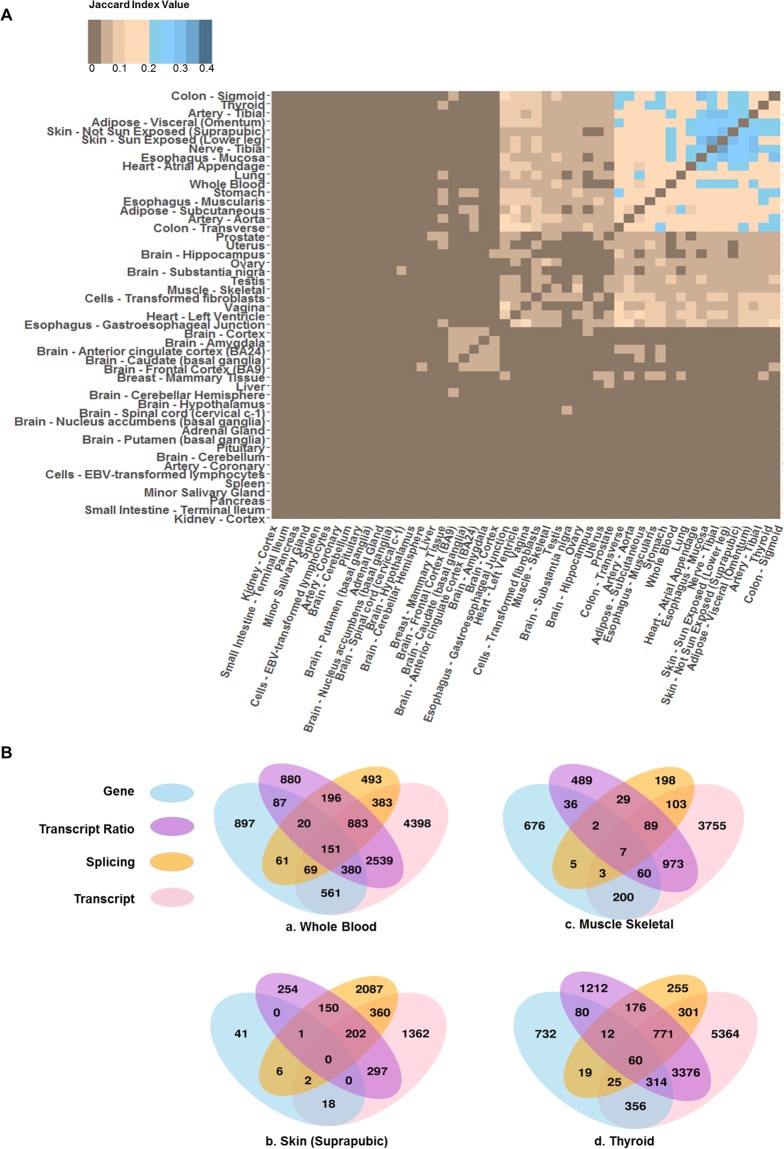
Comparison of age-associated genic changes across tissues and across approaches to capture genic changes. (**A**) Clustering of tissues based on pair-wise similarity of genes affected by age-associated splicing, based on Jaccard Index. The darker blue color indicates higher similarity and darker brown indicates lower similarity. (**B**) Overlap of gene sets affected by age-associated changes detected at the level of gene expression (sky blue), transcript ratio (medium orchid), splicing events (orange) or transcript expression (pink). (a–d) show the data for whole blood, Skin, Muscle-skeletal, and Thyroid tissues respectively, as examples. Data for all tissues are provided in Supplementary Fig. S4.

Alternative splicing has been shown to be tissues-specific^23–25^. Here we assessed the extent to which this is true of age-associated splicing changes. Toward this, we quantified tissue-pair similarity in age-related splicing as the Jaccard index based on the genes involving age-related splicing in the two tissues. As evident in Fig. 3A, most tissues do not share age-associated alternatively spliced genes, implying that such events are tissue-specific, similar to the alternative splicing itself. Hierarchical clustering of tissues based on their pairwise Jaccard index revealed three clusters (Fig. 3A). As expected, Skin – Not Sun Exposed (Suprapubic) and Skin – Sun Exposed (Lower leg) have a high Jaccard index, and so do Colon – Sigmoid and Colon – Transverse. The cluster of 9 tissues (Fig. 3A top right corner), share 19 age-associated alternatively spliced genes (Supplementary Table S2). Interestingly, 15 out of the 19 genes are involved in regulating macromolecule interactions, including binding to proteins, lipids, or nucleic acids.

### Age-related splicing events are potentially functionally linked to ageing process

The 49,869 significant age-associated events across 48 tissues correspond to 9,884 genes. We performed functional term enrichment analysis in these genes in a tissue-specific fashion, using NIH’s David online tool^26,27^. We selected the terms that were enriched (FDR <  = 5%) in at least 3 tissues and performed hierarchical biclustering based on those terms’ enrichment levels (fold change) across tissues (Supplementary Fig S3). We also provide a TreeMap view^28^ of GO terms that are enriched among age-associated spliced genes in at least one tissue in Supplementary Fig. S9. GO annotation is far from complete, noisy, and lacks resolution, and tissue context, making functional interpretation of enriched process in a specific context challenging. Therefore, even though we identified enriched processes in a tissue-specific way, we chose to take a broad look at the enriched processed across tissues, for a more robust interpretation. Supplementary Table S3 lists the top 15 biological functions ranked according to either the number of affected tissues or fold change. These top functional terms include some well-studied processes linked to ageing. For example, mitochondrion and peroxisome and their associated processes are implicated in balancing the levels of reactive oxygen species in the cell^29^, and cell-cell adhesion is essential for mediating tissue integrity and stem cell niche^30^. Ribosome and ribosomal ribonucleoprotein were ranked among the top by both measures, which is in agreement with the emerging view that the ability of cells to maintain a healthy and relatively stable pool of proteins under continuous stresses that accumulate over time is a major determinant of lifespan (Andrew Dillin Cell Meta 2016). Interestingly, genes with age-associated splicing in Muscle – Skeletal (358 genes), Whole Blood (2,073 genes) and Adipose-Subcutaneous tissues (1,143 genes) are linked with all top enriched processes, with very little overlap among the respective gene sets. The interaction between aging process and alternative splicing can be bi-directional, that is, many age-related events may be downstream effects, rather than causes, of ageing. The genes that are involved in typical aging-related biological functions, as well as exhibit age-associated splicing patterns may contribute to aging and aging related phenotypes, while other genes that although exhibit age-associated splicing pattern but otherwise are not involved in ageing-related processes may represent downstream effects. Overall, these results show that in some tissues age-related splicing events may be functionally linked to the phenotypic changes associated with ageing, while they may be the downstream effect of ageing process in others. In addition, 78.6% of overall biological processes (include most ageing related processes) are recaptured by only performing gene ontology analysis on genes uniquely associated with splicing changes, which implies that these reported ageing related process may be related to age associated splicing instead of gene expression.

### A focus on splicing uniquely reveals numerous age-associated genes

Both transcriptional and splicing processes can change with age. With regards to splicing regulation, while the age-associate changes must be mediated at the level of individual splicing events, the downstream effects of these changes on the age-related phenotypes are mediated by changes in the levels of specific transcripts. Toward obtaining a global view of age-associated changes in these various aspects of the transcriptome, analogous to splicing event-based model above, we implemented linear models to detect age-associated changes in gene expression, transcript expression, and relative transcript ratios (Methods), and compared the genes corresponding to the significant age-associated events in the four categories – individual splicing events, gene expression, transcript expression, and relative transcript usage, shown in Fig. 3B, for 4 select tissues (all tissue results are provided in Supplementary Fig. S4). It is apparent from this result that a focus on splicing and transcripts uniquely reveals numerous age-associated genes. We have included these unique age-associated genes in Supplementary data 1. Specifically, for instance, 18% of the metabolic genes, known to be significant ageing markers, are revealed as age-associated only at the transcript level, and not at the level of overall gene expression, e.g., Phosphofructokinase gene locus (PFK), which has previously been targeted in cancer therapy^31^, exhibits age-associated changes at the transcript level in 21 tissues but not at the level of gene expression (Supplementary Fig. S5 illustrates the known isoforms of PFK and their age-associations^32^). These results suggest that ageing process has substantial association with post-transcriptional regulation beyond its known associations with transcriptional processes.

### A splicing-based model is informative of biological and cellular age

**Figure 4:**
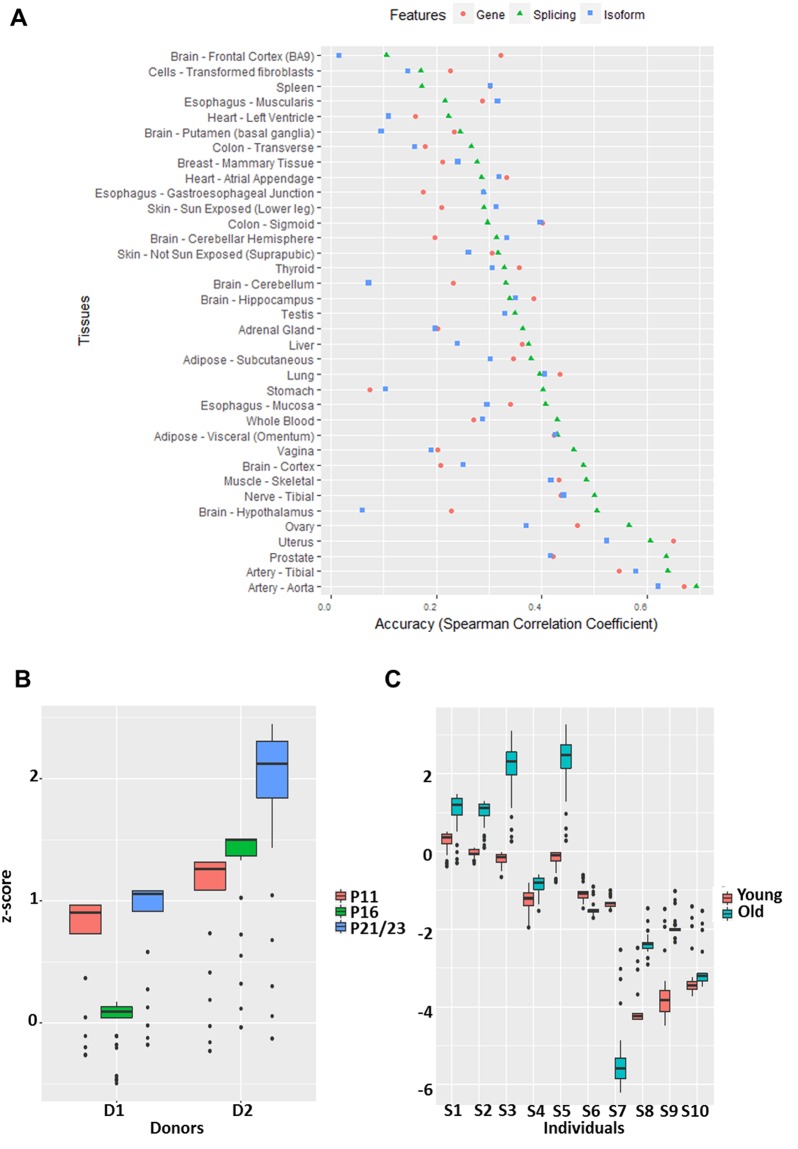
(**A**) Accuracies of prediction of age using models based on gene expression (pink circle), splicing events (green triangle) and isoform expression (blue square) across tissues. The accuracy is measured by Spearman correlation between predicted and true ages based on cross-validation. (**B**) The figure shows predicted relative ages for two sets of skin fibroblast cell culture (D1 and D2) across three passages (PX: passage X). (**C**) The figure shows predicted relative ages of ten pairs (from individuals S1 through S10) of longitudinal skin fibroblast samples. The matched young and old samples were derived from the same individual over time. The y-axis in (**B**,**C**) denote normalized predicted age.

We first assessed the extent to which genome-wide splicing profile in an individual is reflective of the individual’s biological age. Furthermore, to compare the merits of splicing profile relative to gene expression and transcription expression profiles, we constructed three analogous models of age based on splicing profile, gene expression profile, and transcript expression profile (Methods). The accuracy was quantified as the Spearman correlation between predicted ages and true ages in cross-validation samples. Figure 4A shows the 10-fold cross-validation prediction accuracies of the three models across 36 tissues; only the tissues in which all three models yielded positive predictive accuracy are shown. A direct comparison of model accuracies based on paired Wilcoxon test across tissues reveals that splicing-based model outperforms the other two models (p-values <  = 0.05). Surprisingly, the isoform-based model is not significantly better than the gene expression-based model, which may be due to incompleteness and inaccuracies in isoform annotations and noisy quantification of isoform expression. In addition, we implemented an alternative approach to estimate accuracy. We partitioned the individuals into two classes of old and young (Old class: the oldest 25% and Young class: the youngest 25%) and performed a standard classification based on Lasso regression. The results are consistent, in that the splicing events results in better prediction accuracy than the other two modalities, and on average the prediction accuracy is 71% (Supplementary Fig. S6). Overall our results suggest that global splicing profile is more predictive of age compared to gene and isoform expression. We also compared the 7 types of splicing events regarding their individual ability to predict age following an analogous procedure as above. The results are shown for the 25 tissues in which all seven models yielded positive predictive accuracies (Supplementary Fig. S7). Overall the exon-skipping events are the best predictor of age compared to the other 6 types of events (all Wilcoxon test p-values < = 7.4e-3).

Next, we assessed whether a splicing-based model of age constructed using GTEx skin fibroblast samples can successfully predict relative ages of two independent longitudinal datasets of skin fibroblast (Methods). This analysis is limited by the data availability. The first dataset consists of cell passage (a standard proxy for cellular age) data, which includes young (11 passages), middle (16 passages) and old samples (21 or 20 Passages) for two healthy individual derived skin fibroblasts (6 samples). In addition, donor 1 is younger than donor 2. The second dataset^33^ includes 10 pairs of longitudinal samples from 10 donors at two different ages separated by 15.7 years on average (20 samples). To specifically assess the contributions of age-associated splicing events, the model was constructed using only the significant age-associated splicing events detected in GTEx (Methods).

In the first validation dataset (Fig. 4B), our GTEx-trained model correctly predicts the lowest passage cells to be younger than the oldest passage cells in donor 1 (D1), but fails to correctly predict the age of middle passage cells. However, in donor 2 our model correctly predicts the relative ages of the three cell passages. Out of total 19 pairwise comparisons (based on donors’ age and cellular age), we correctly order the samples in 16 (84%) of all the cases. A paired Wilcoxon test of the 19 pairwise predicted ages showed significance with p-value is 0.0047. In the second longitudinal dataset (Fig. 4C), in 8 out of 10 cases, our model correctly predicts the relative ages of the two samples from the same individual.

### Some of the age-associated splicing events may be driven by age-associated expression changes in the upstream splice factors

**Figure 5:**
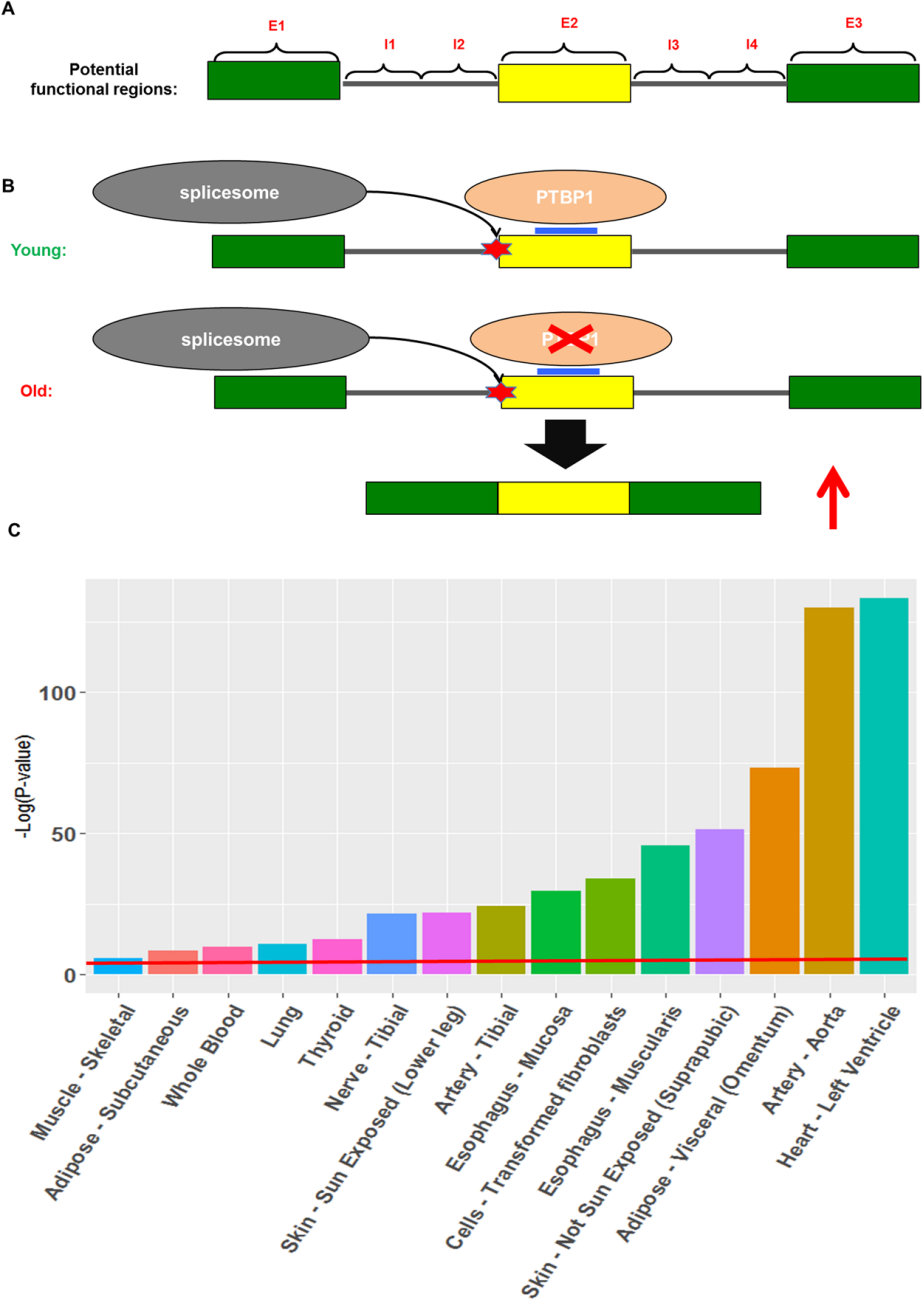
(**A**) The 7 functional regions relative to an exon (yellow rectangle) potentially impacting the splicing event. (**B**) Illustration of a potential mechanism of age-associated change in exon inclusion whereby the expression of a splicing factor PTBP1, which suppresses the inclusion of the cassette exon, decreases with age, thereby resulting in an increase in exon inclusion with age. (**C**) Additional contribution of splicing events to the explained variance of Hypertension, in multiple tissues shown on x-axis. The y-axis denotes the significance (-log(p-value)) based on a log-likelihood ratio test. The red line indicates p-value = 0.05.

In exploring the mechanisms underlying age-associated splicing changes, we assessed whether certain motifs near the splicing event recognized by a splicing factor, along with age-associated changes in the expression level of the splicing factor, can together explain the changes in splicing. This analysis was restricted to exon skipping events. In each tissue independently, using the significant tissue-specific events, separately for up-regulated and down-regulated event, we identified the splicing regulators whose RNA-recognition motifs (obtained from^34^) were significantly enriched in any of the 7 regions near the cassette exon (Fig. 5A), relative to the background cassette exons whose usage did not vary with age (Methods). An enrichment threshold (FDR <  = 0.1) was applied to retained potential functional motifs.

Supplementary Table S4 lists the 9 potential splicing factor drivers of age-associated splicing changes identified in skin fibroblast. Splice factor PTBP1 is known to inhibit exon retention by binding to exonic splicing enhancers^35^. We found that PTBP1 motifs are significantly enriched within the middle exon among up-regulated exon inclusion events and consistently, PTBP1 expression showed a significant decrease with age (standardized age covariate coefficient = −35.8). Illustrated in Fig. 5B, this example suggests a potential mechanism whereby an age-associated decrease in PTBP1 concentration lifts its inhibitory effect resulting in increased exon retention at multiple loci.

We sought for experimental support for splicing factor-mediated changes in splicing through age. We obtained 3546 potential PTBP1 targets in HeLa cell based on PTBP1 CLIP-seq data^36,37^; such data is not available for skin. We then independently, using our approach, identified 46 genes whose age-associated splicing is potentially a downstream effect of PTBP1. We found that experimentally identified potential target genes of PTBP1 are highly enriched among the targets identified by our pipeline (Fisher test p-value = 1.3E-05; Odds-ratio = 2.4).

### Age-associated splicing contributes to complex age-related diseases

Several complex diseases, many of which exhibit increased incidence with age, have been shown to be associated with distinctive tissue-specific gene expression profiles^38,39^. Potential mechanisms linking alternative splicing to age-related diseases have been explored previously. Alternative splicing might change the transcript ratio leading to a greater fraction of impaired protein isoform, truncated wild type protein, or suboptimal isoform ratios, which might affect the cellular processes underlying age-related diseases^40^. Alternative splicing within genes EAAT2, SALL1 and TAU have been shown to contribute to age-related diseases^40–43^. Given our observed links between splicing and ageing, we assessed the extent to which tissue-specific splicing profile potentially contributes to age-related diseases. We tested this for 4 diseases, including hypertension, for which there is a sufficient number of samples in GTEx in multiple tissues. For a given disease and tissue, Log Likelihood Ratio (LLR) test (Method) was used to assess the independent contribution of splicing profile to the disease by controlling for age, gender, and gene expression. As shown in Fig. 5C, relative to gene expression, age and gender, splicing can significantly (p-value < = 0.05) explain additional hypertension disease state variance in all of the 15 tissues tested. The three most significant tissues are Heart, Artery, and Adipose, which have well-established mechanistic links to hypertension [REF]. Results for three additional pathologies – Heart Attack, Chronic Respiratory Disease, and Diabetes mellitus type II, show consistent results (Supplementary Fig. S8). Due to relatively small sample size, we analyzed fewer tissues for these three diseases. In 7, 4 and 3 tissues respectively for Diabetes mellitus type II, Chronic Respiratory Disease and Heart Attack, age-associated splicing events provide significant independent contribution in addition to age, gender and gene expression. In addition, we show that our results are robust to potential confounding by human ancestry (race) by additionally controlling for race in both the null and alternative models (concordance correlation coefficient of the two p-value distributions is 0.98). These results suggest links between splicing and complex age-related diseases independent of age and the genome-wide gene expression profile.

## Discussion

Overall, exploiting ~8,500 tissue-specific transcriptomes in 544 individuals, we identified 49,869 age-related splicing events for 7 distinct types of splicing events across 48 tissues. In contrast to previous related works, our model stringently controls for potential hidden confounding factors. In addition to validating our splicing-based model of age in independent longitudinal and cell passage datasets, we show that splicing profiles are a better predictor of biological age than gene and transcript expression levels alone, and the splicing profile provides an independent contribution to age-related complex diseases. Finally, we propose a potential mechanism underlying age-associated splicing changes mediated by a concomitant change in the expression level of the upstream regulatory splice factor.

Mazin *et al*. identified 3,132 and 6,114 significant age-related splicing events in the two brain regions respectively, with 1,484 events in common, which represents ~5% of all events assessed. In contrast, we identified 1,066 events from the same regions. However, these represent ~0.06% of all events that we assessed, potentially reflecting the stringency of our approach. A direct comparison of events detected by their results and ours could not be made because of incompatibility of event definition. These differences could potentially be attributed to multiple factors related to sample sizes and controls.

Besides age-associated splicing studies mentioned above, recently, Yang *et al*. reported age-associated gene expression changes across 7 tissues from GTEx version 4^9^, and found that Blood has the most age-related gene expression changes, consistent with our splicing-based results. Lung, Muscle and Heart tissues were also shown to have significant age-associated changes in both our studies. Our study however uniquely identifies Skin to have a large number of age-associated splicing changes, which may suggest that age-associated effects in skin primarily affect splicing levels and are not reflected in gene expression levels. However, broadly, the genes revealed by both our and previous studies are related to common ageing-related biological processes such as mitochondrial function, DNA repair, Cell Cycle, ATP-binding, etc. in Whole Blood, Muscle and Heart tissues.

Anomalous gene expression is often the first major factor considered when investigating ageing and complex age-related diseases. However, our study suggests that tissue-specific splicing profiles may provide an additional contribution to ageing and age-related diseases. Indeed previous studies have directly linked splicing dysregulation to diseases, independent of gene expression^36^.

As the first multi-tissue study of age-associated splicing changes, we were able to compare such changes across tissues. Our observed lack of cross-tissue commonality is consistent with previous studies suggesting that the alternative splicing, as well as gene expression regulation, are highly tissue-specific^44^, and tissue-specific changes in the expression and splicing regulators can explain tissue-specificity of the age-related splicing changes.

Importantly, our analysis suggests one potential mechanism of age-associated splicing changes, namely, via age-associated expression changes of splicing regulators. Given the links between splicing and transcription^45,46^, it is conceivable that several other transcriptional mechanisms can contribute to age-related splicing changes. For instance, age-related changes in DNA methylation and histone modifications have been previously reported^47,48^. Specifically, DNA methylation has been shown to be excellent an biomarker of age^49^. Polymorphisms can also affect age-associated splicing changes, which may in turn manifest in variable vulnerability to age-related diseases. Our study provides a methodological framework and resource for future targeted investigation of links between splicing and ageing.

## Method

### Splicing Level Quantification using GTEx data

The processed transcript expression data for ~8500 samples from 544 donors across 48 tissues were downloaded from Genotype-Tissue Expression (GTEx) database version 6^13^. GENCODE genome annotation version 19^16^ and SUPPA software package^50^ was employed to extract 7 types of exon-centric splicing event annotations (exon skipping, alternative 5’, alternative 3’, mutually exclusive exons, alternative first exon, alternative last exon, intron retention). Then in each sample SUPPA was used to quantify the splicing level of each annotated event in terms of PSI values (Percent Splicing Index).

### Model for detecting age-associated splicing events

To detect significant age-associated splicing events, we modeled the association between each event and age across multiple samples as follows:

1 $$PS{I}_{ij}={\alpha }_{i}+{\beta }_{i}^{1}AG{E}_{j}+{\beta }_{i}^{2}GENDE{R}_{j}+\sum _{k=1}^{n}{\beta }_{i}^{k+2}{\rm{PEER}}({{\rm{CF}}}_{{\rm{j}}}^{{\rm{k}}})+{\varepsilon }_{ij}$$

where *PSI*_*ij*_ is the splicing level for event i in sample j, *AGE*_*j*_ and *GENDER*_*j*_ denote the age and gender of individual j respectively, PEER $({{\rm{CF}}}_{{\rm{j}}}^{{\rm{k}}})$ denotes the k^th^ confounding factor estimated using PEER packages^51^ for individual j. α_*i*_ is the intercept for the model of event i, ${\beta }_{i}^{1}$ and ${\beta }_{i}^{2}$ are the coefficients respectively for age and gender covariate for event i, ${\beta }_{i}^{k+2}$ is the coefficient of the kth confounding factor for event i, ${\varepsilon }_{ij}$ is the error in the model for event i of individual j. In addition, in this model *n* is the number of hidden confounding factors we estimated (n = 20) compared to 15 hidden confounding factors used in Brinkmeyer-Langford *et al*.'s age-associated gene expression study^52^.

Since some genes are not expressed in some of the samples, when modeling such splicing events corresponding to those genes, we excluded samples where the gene was not expressed (reported as −1 by SUPPA package). Further, to ensure statistical power, we only analyzed events having at least 50 samples where the corresponding gene had non-zero expression. For each event, we fitted the data to the model and examined the age covariate coefficient ${\beta }_{i}^{1}$, and assessed the significance for its deviation from zero, and applied FDR control across all tested events. In addition, we performed permutation test by shuffling the age distribution across all individuals. For each event, permutation test is performed for 1000 times and estimate the significance of the age covariate. Events with FDR < = 0.05 and fewer than 5% of the permuted data showing significance (p-value < = 0.05) were deemed significanctly age-associated.

### Correcting for confounding factors using PEER package

We ensured that our detected link between an event and age is not due to confounding factors, as follows. PEER software package is widely used in eQTL studies to correct for potential hidden confounding factors such as batch effects^51^. For each tissue, given the global PSI profiles (including all events of all types) for all individuals, we estimated 20 ‘PEER’ factors. Then we estimated pearson correlation between each PEER factor and age across all individuals, and excluded the factors, in an event-specific way, that were significantly correlated with age (P < 0.05).

### Functional Enrichement analysis

We map each significant age-assocaited splicing event to its corresponding gene, and identifed significantly enriched (FDR < = 0.05) GO terms in a tissue specific manner using NIH’s David online tool^26,27^. We further retained only the terms that were enrcihed in at least three tissues. Finally we performed hierarchical biclustering based on the enrichment level (fold change) of enriched functional terms across tissues. In addition, we used package “REVIGO”^28^ to generate a TreeMap view of the enrcihed GO terms, and for each GO term the number of tissues in which it was significantly enriched (FDR < = 0.05) was used as the enrichment score for visualization.

### Cross tissue similarity in age-associated splicing

Jaccard index is a metric to measure the similarity between two sets ($J(A,B)=\frac{(A{\cap }^{}B)}{(A{\cup }^{}B)}(0\le J(A,B)\le 1)$). We employed this metric to measure the similarity of age-associated splicing between two tissues. For each tissue, we identified the genes having at least one age-associated splicing event, and estimated the Jaccard index using the tissue-specific gene sets.

### Age associated gene, isoform and isoform ratio detection across tissues

In order to assess age-associated changes in gene expression, isoform expression, and isoform ratio levels, we developed three linear models.

2 $${\rm{Gene}}\,{\rm{model}}:{G}_{ij}={\alpha }_{ij}+{\beta }_{i}^{1}AG{E}_{j}+{\beta }_{i}^{2}GENDE{R}_{j}+\sum _{k=1}^{n}{\beta }_{i}^{k+2}{\rm{PEER}}({{\rm{CFG}}}_{{\rm{j}}}^{{\rm{k}}})+{\varepsilon }_{ij}$$

3 $${\rm{Transcript}}\,{\rm{model}}:{T}_{ij}={\alpha }_{ij}+{\beta }_{i}^{1}AG{E}_{j}+{\beta }_{i}^{2}GENDE{R}_{j}+\sum _{k=1}^{n}{\beta }_{i}^{k+2}{\rm{PEER}}({{\rm{CFT}}}_{{\rm{j}}}^{{\rm{k}}})+{\varepsilon }_{ij}$$

4 $${\rm{Transcript}}\,{\rm{ratio}}\,{\rm{model}}:T{R}_{ij}={\alpha }_{ij}+{\beta }_{i}^{1}AG{E}_{j}+{\beta }_{i}^{2}GENDE{R}_{j}+\sum _{k=1}^{n}{\beta }_{i}^{k+2}{\rm{PEER}}({{\rm{CFT}}}_{{\rm{j}}}^{{\rm{k}}})+{\varepsilon }_{ij}$$

i and j represent an event and an individual respectively, and n denotes the number of confounding PEER factors considerd. ${\beta }_{i}^{1},{\beta }_{i}^{2}and{\beta }_{i}^{k+2}$ denote the coefficient for age, gender and kth confounding factor for event i of individual j. G denotes gene expression, T represents for transcript expression and TR denotes transcript ratio $(\frac{{T}_{x}}{{\sum }^{}{T}_{x}})$. CFG (equation 2) and CFT (equation 3 and 4) denote confounding factors derived from the genome-wide gene expression and transcript expression profiles respectively. The procedure is the same as that was used to detect age-associated splicing events.

### Predicting Age using splicing level, isoform expression, and gene expression

To compare the power of splicing level, isoform expression and gene expression in predicting age, we built a LASSO regression model for each of them. For the splicing model, MDS analysis was performed over the population PSI values, and top 30 PCs were used as features in the linear model to predict age. In order to remove sampling bias, we performed randomized 10-fold cross validation 100 times and estimate the average cross-validation predicted age for each sample, and estimate the accuracy as the Spearman correlation between the predicted and the given age. For comparison, we implemented an identical procedure using gene-level expression as well as isoform-level expression.

### Predicting relative ages in independent datasets using splicing-based model of age

To validate our splicing-based model of relative age, we build a lasso regression model based on our detected top age-associated splicing events in GTEx data to predict the relative ages in longitudial data and celluar age data. Recall that in detecting significant age-associated splicing events, we perform a permutation test. Since the permutation is stochatsic, we repeated it 10 times and selected 141 age-associated events detected in at least 8 permutations. These 141 events were then used to build a model of age in GTEx data, which is used to estimate the age of each sample in the independent validation set. The predicted age is transformed into a z-score using the predicted age distribution of GTEx data ro represent the relative ages. 100 rounds of model fitting were performed to remove sampling bias (the penalty parameter lambda was optimized based on randomized cross-validation) to generate a distribution of predicted z-scores for each sample in the independent dataset. Then Wilcoxon tests were performed to compare the relative ages of two samples.

### Detecting potential upstream drivers of age-associated splicing changes

Here the goal was to test the hypothesis that the age-associated change in expression of the upstream splicing factor gene contribute to the downstream age-associated splicing changes. We obtained 121 experimentally valided RNA motifs mediating splicing (Supplementary Table S4), for which the corresponding splicing factors are also known. All significant age-assocaited exon skipping events from skin fibroblast tissue were divided to 3 classes based on the direction of their age-associate change (class 1: increase with age, class 2: stable, class 3: decrease with age). We performed motif enrichment analysis between class 1 and 2, and also between 2 and 3. More specifically, the frequency of each motif between two classes was compared using wilcoxon test, and FDR control was applied to select siginifcantly enriched motifs.

Next, we performed differential gene expression (transcript level) analysis across ageing using a model analogous to the model for splicing above, and detected the splicing factors (i) whose gene expression (transcript level) is significantly (p-value < = 0.05) associated with age, and (ii) whose motifs are enriched near the age-associated splicing events.

### Estimating contribution of splicing profile to complex diseases

We build two nested linear models of age-related diseases in the GTEx population independetly in each of the 48 tissues. The first ‘*null’* model (Equation 5) relates the binary disease state to age (AGE), gender (GENDER), and gene information (GE), and the second ‘*splicing*’ model (Equation 6) additionally uses splicing information; however, we only included significant age-associated splicing events.

5 $$Null\,model:{D}_{j}=\alpha +{\beta }^{1}AG{E}_{j}+{\beta }^{2}GENDE{R}_{j}+{\beta }^{3}G{E}_{j}$$

6 $$Splicing\,model:{D}_{j}=\alpha +{\beta }^{1}AG{E}_{j}+{\beta }^{2}GENDE{R}_{j}+{\beta }^{3}G{E}_{j}+{\beta }^{4}PS{I}_{j}$$

*D*_*j*_ denotes dieases status (0: normal, 1: disease). For both gene and splicing information, we reduced the dimensionality by performing MDS analysis using the top 100 PCs in both cases. Given the two model fits, we estimated the Log-Likelihood ratio and estimated the contribution of splicing information using Chi-sqaure test.

## Electronic supplementary material

Supplementary Data 1

Supplementary Information
