# Supplementary material for: Comprehensive map of age-associated splicing changes across human tissues and their contributions to age-associated diseases
Source: Sci Rep. 2018 Jul 19;8:10929. doi: 10.1038/s41598-018-29086-2 (PMC6053367; doi:10.1038/s41598-018-29086-2)
Supplement: Supplementary file 2 — Supplementary Information [file 41598_2018_29086_MOESM2_ESM.pdf]

Comprehensive map of age-associated splicing changes across human tissues and their contributions to age-associated diseases

Kun Wang[1, 2], Di Wu[2], Haoyue Zhang[2], Avinash Das[1], Mahashweta Basu[1], Justin Malin[3], Kan Cao[2, *], Sridhar Hannenhalli[1, 2, *]

[1]Center for Bioinformatics and Computational Biology, University of Maryland, College Park, MD, 20742, USA

[2]Department of Cell Biology and Molecular Genetics, University of Maryland, College Park, MD, 20742, USA

[3] Computational Biology Branch, National Center for Biotechnology Information, National Institutes of Health, Bethesda, Maryland 20892

*Corresponding author

sridhar@umiacs.umd.edu

kcao@umd.edu

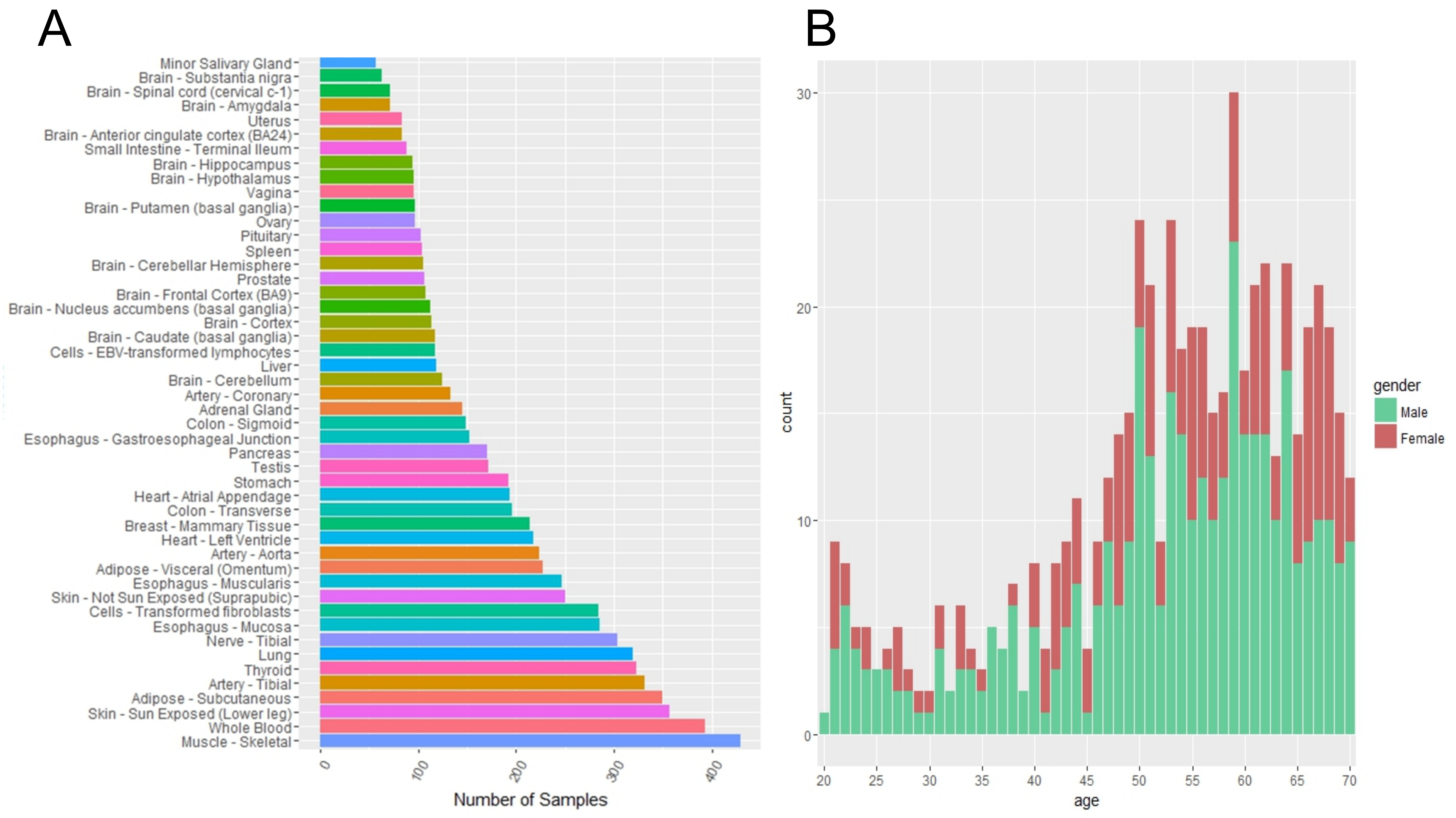

Figure S1: Summary of GTEx data. (A) Number of samples across 54 tissues. (B) Distribution of sample age and gender.

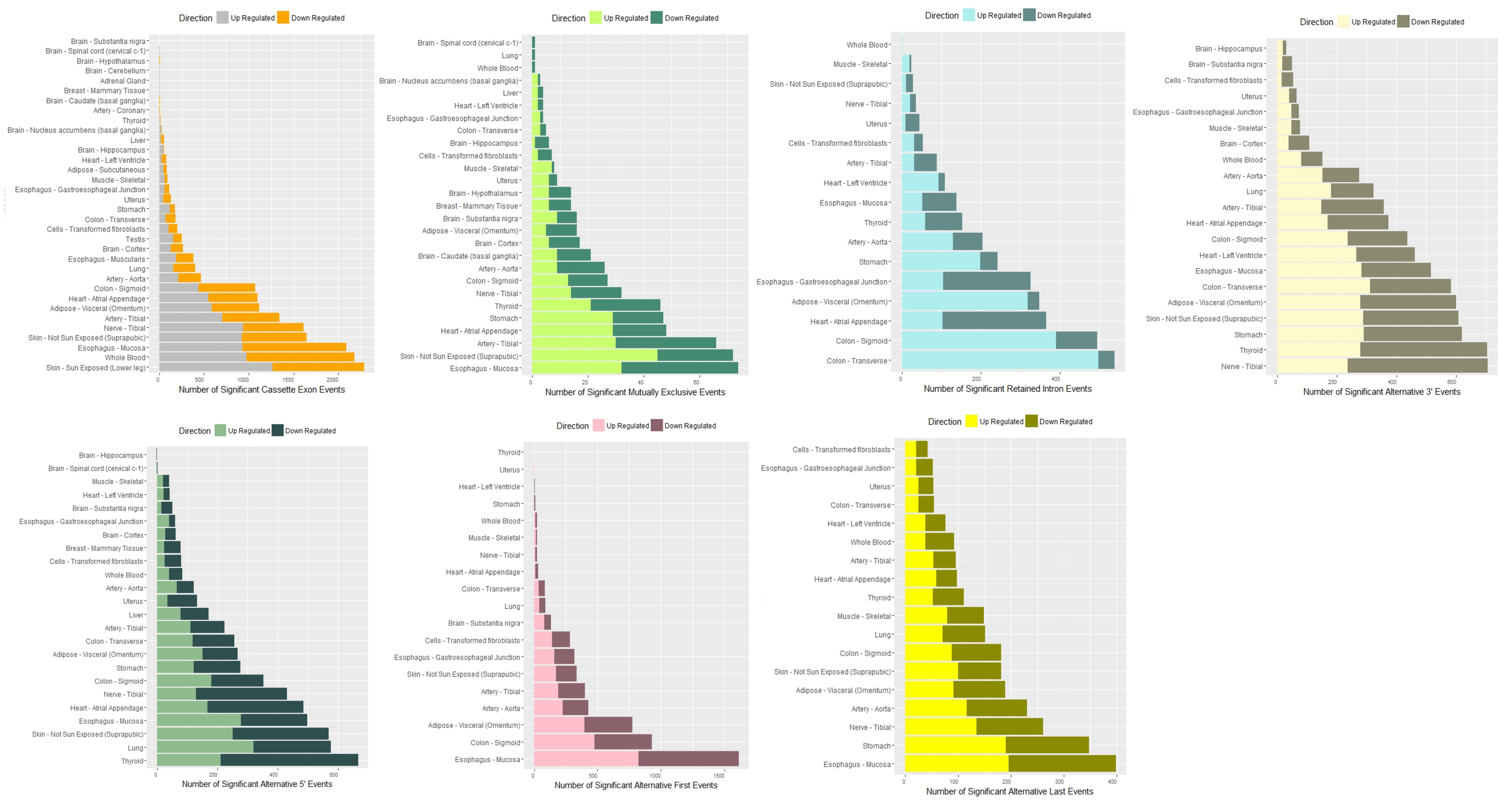

Figure S2: Number of significant up and down-regulated splicing events across tissues for the 7 types of splicing events.

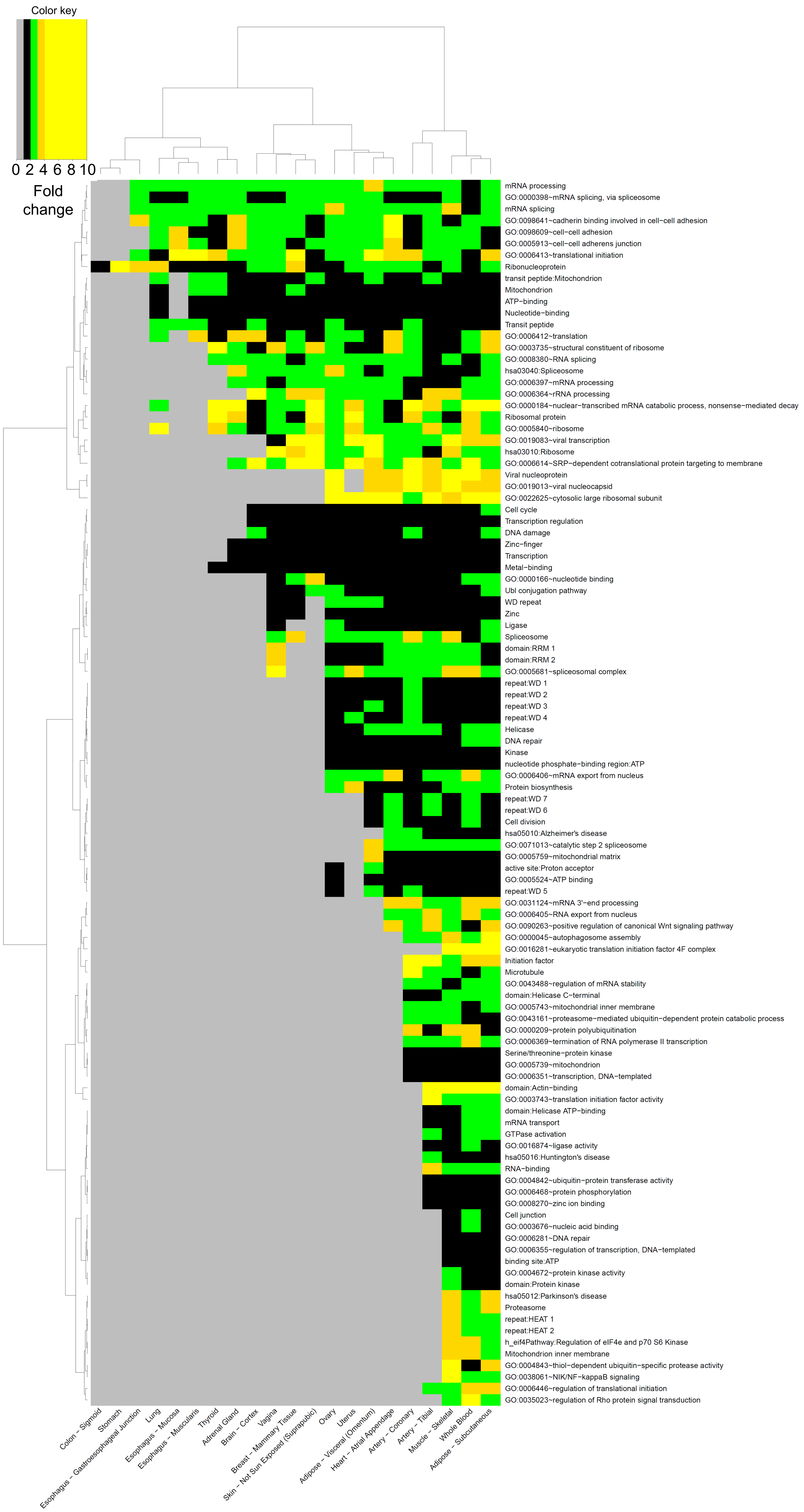

Figure S3: Functional enrichment among genes affected by age-associated splicing across tissues. Columns correspond to tissues, rows to biological functions, colors indicate fold-change.

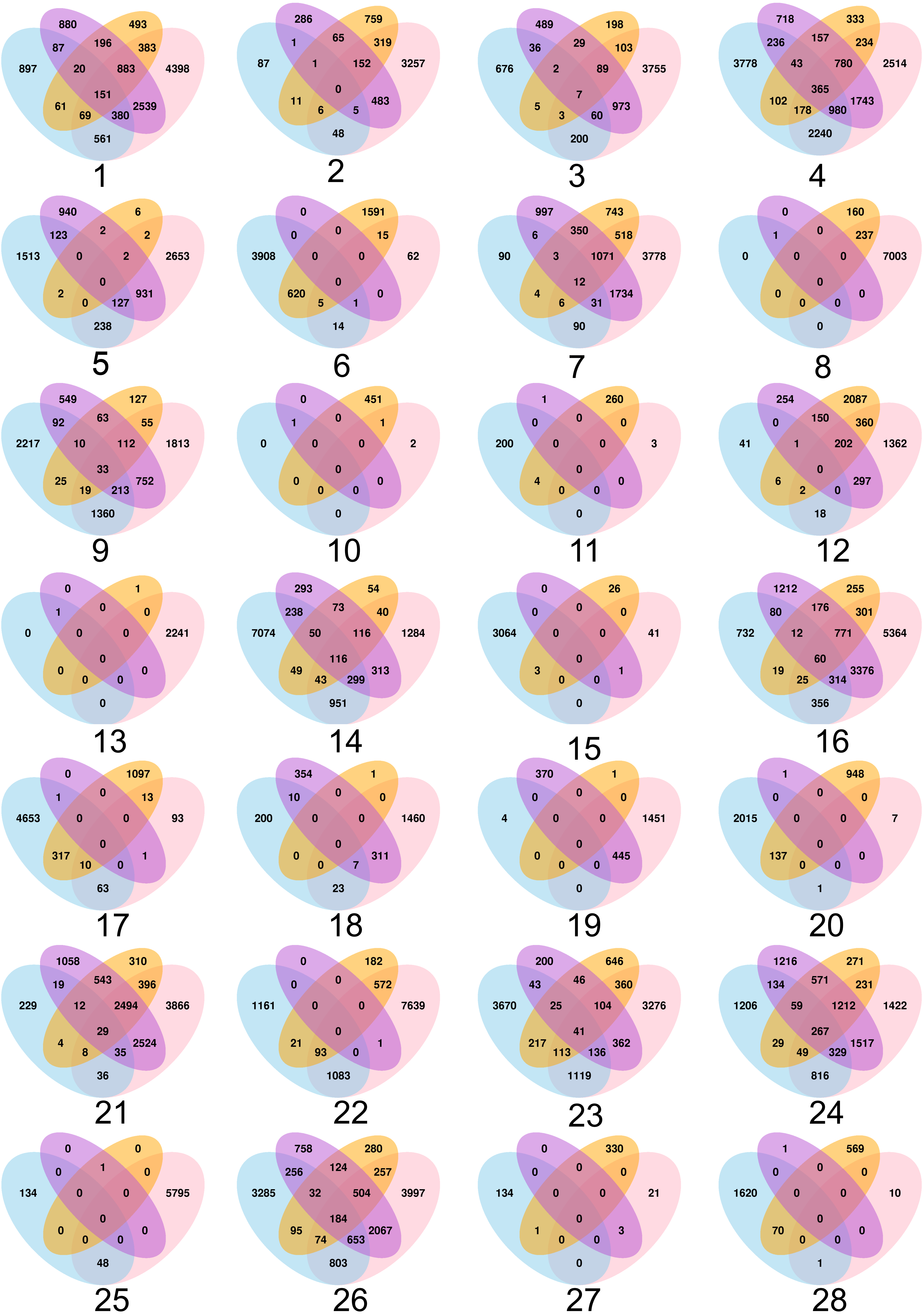
880 493 87 196 383 897 20 883 4398 151 61 2539 69 380 561
1
286 759 1 65 319 87 1 152 3257 0 11 483 6 5 48
2
489 198 36 29 103 676 2 89 3755 7 5 973 3 60 200
3
718 333 236 157 234 3778 43 780 2514 365 102 1743 178 980 2240
4
940 6 123 2 2 1513 0 2 2653 0 2 931 0 127 238
5
0 1591 0 0 15 3908 0 0 62 0 620 0 5 1 14
6
997 743 6 350 518 90 3 1071 3778 12 4 1734 6 31 90
7
0 160 1 0 237 0 0 0 7003 0 0 0 0 0 0
8
549 127 92 63 55 2217 10 112 1813 33 25 752 19 213 1360
9
0 451 1 0 1 0 0 0 2 0 0 0 0 0 0
10
1 260 0 0 0 200 0 0 3 0 4 0 0 0 0
11
254 2087 0 150 360 41 1 202 1362 0 6 297 2 0 18
12
0 1 1 0 0 0 0 0 2241 0 0 0 0 0 0
13
293 54 238 73 40 7074 50 116 1284 116 49 313 43 299 951
14
0 26 0 0 0 3064 0 0 41 0 3 1 0 0 0
15
1212 255 80 176 301 732 12 771 5364 60 19 3376 25 314 356
16
0 1097 1 0 13 4653 0 0 93 0 317 1 10 0 63
17
354 1 10 0 0 200 0 0 1460 0 0 311 0 7 23
18
370 1 0 0 0 4 0 0 1451 0 0 445 0 0 0
19
1 948 0 0 0 2015 0 0 7 0 137 0 0 0 1
20
1058 310 19 543 396 229 12 2494 3866 29 4 2524 8 35 36
21
0 182 0 0 572 1161 0 0 7639 0 21 1 93 0 1083
22
200 646 43 46 360 3670 25 104 3276 41 217 362 113 136 1119
23
1216 271 134 571 231 1206 59 1212 1422 267 29 1517 49 329 816
24
0 0 0 1 0 134 0 0 5795 0 0 0 0 0 48
25
758 280 256 124 257 3285 32 504 3997 184 95 2067 74 653 803
26
0 330 0 0 0 134 0 0 21 0 1 3 0 0 0
27
1 569 0 0 0 1620 0 0 10 0 70 0 0 0 1
28

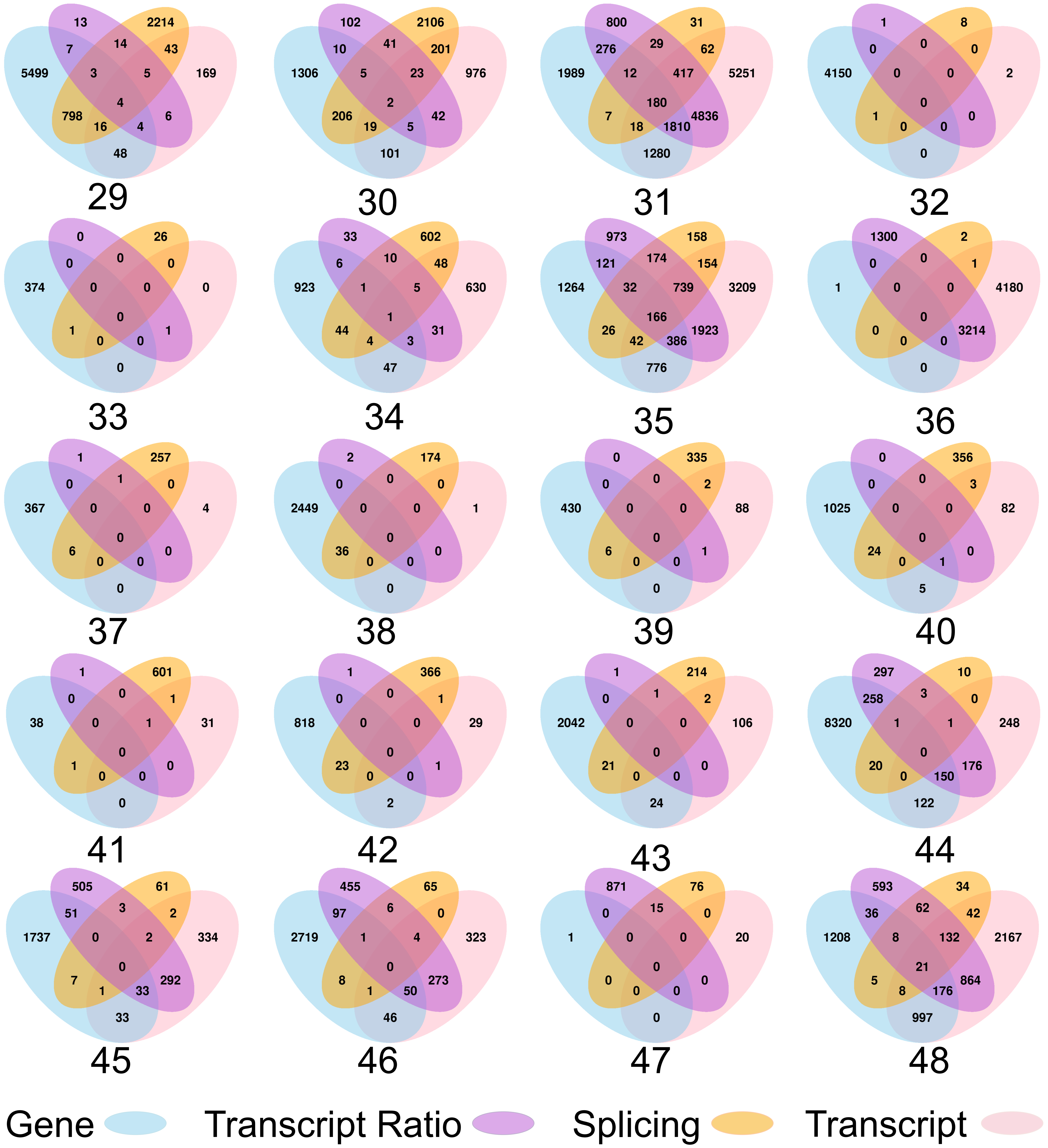

Figure S4: Overlap of gene sets affected by age-associated changes detected at the level of gene expression (sky blue), transcript ratio (medium orchid), splicing events (orange) or transcript expression (pink). (1) – (48) show the data for Whole Blood,Adipose - Subcutaneous,Muscle - Skeletal,Artery - Tibial,Artery - Coronary,Heart - Atrial Appendage,Adipose - Visceral (Omentum),Ovary,Uterus,Vagina,Breast - Mammary Tissue,Skin - Not Sun Exposed (Suprapubic), Minor Salivary Gland,Brain - Cortex,Adrenal Gland,Thyroid,Lung,Spleen,Pancreas,Esophagus - Muscularis,Esophagus - Mucosa,Esophagus - Gastroesophageal Junction,Stomach,Colon - Sigmoid,Small Intestine - Terminal Ileum,Colon - Transverse,Prostate,Testis,Skin - Sun Exposed (Lower leg),Nerve - Tibial,Heart - Left Ventricle,Pituitary,Brain - Cerebellum,Cells - Transformed fibroblasts,Artery - Aorta,Cells - EBV-transformed lymphocytes,Liver,Brain - Hippocampus,Brain - Substantia nigra,Brain - Anterior cingulate cortex (BA24),Brain - Frontal Cortex (BA9),Brain - Cerebellar Hemisphere,Brain - Caudate (basal ganglia),Brain - Nucleus accumbens (basal ganglia),Brain - Putamen (basal ganglia),Brain - Hypothalamus,Brain - Spinal cord (cervical c-1) and Brain – Amygdala respectively.

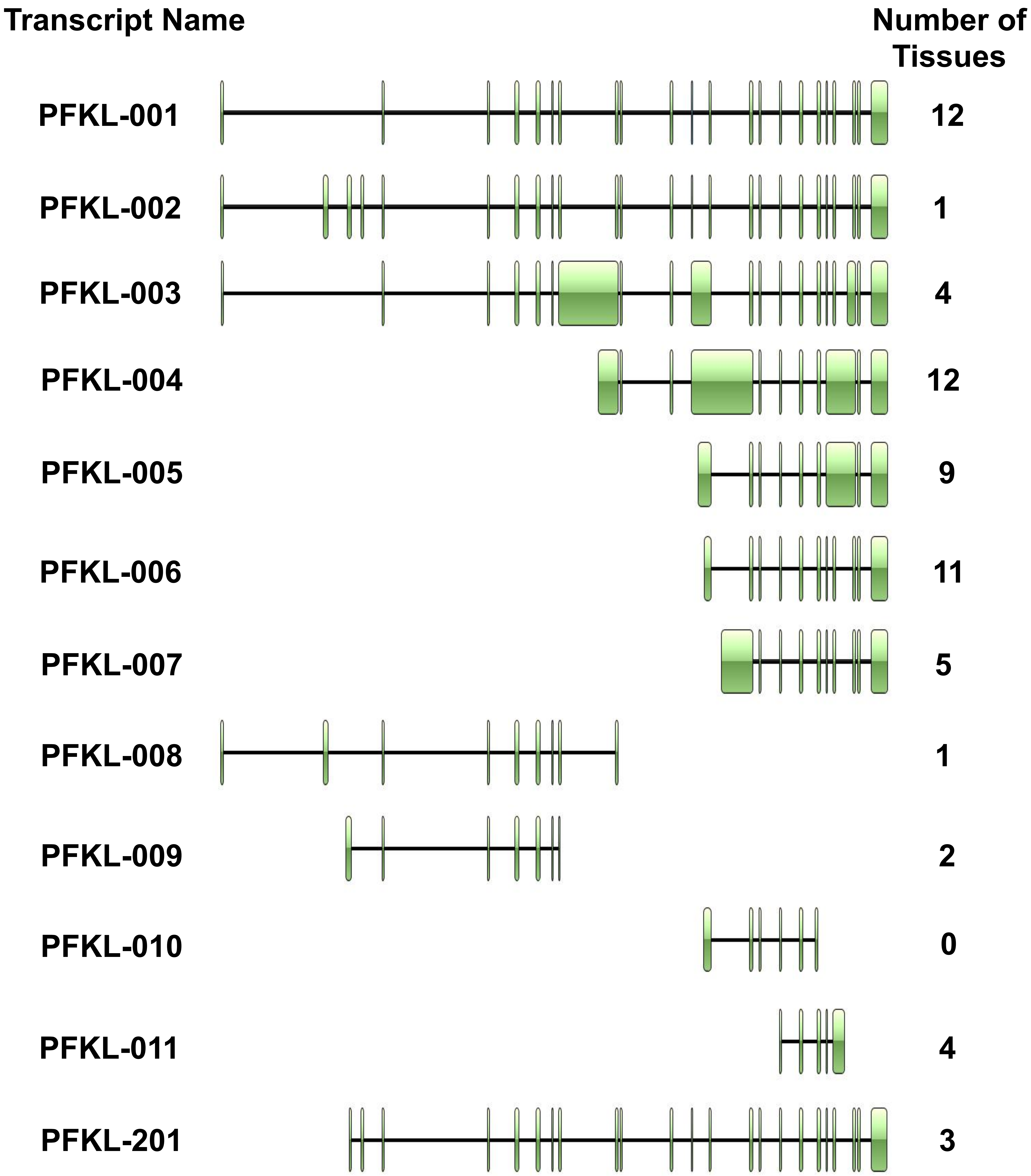

Figure S5: The annotated 12 transcripts of PFKL gene and the number of tissues in which each transcript's expression/ratio changes significantly with age.

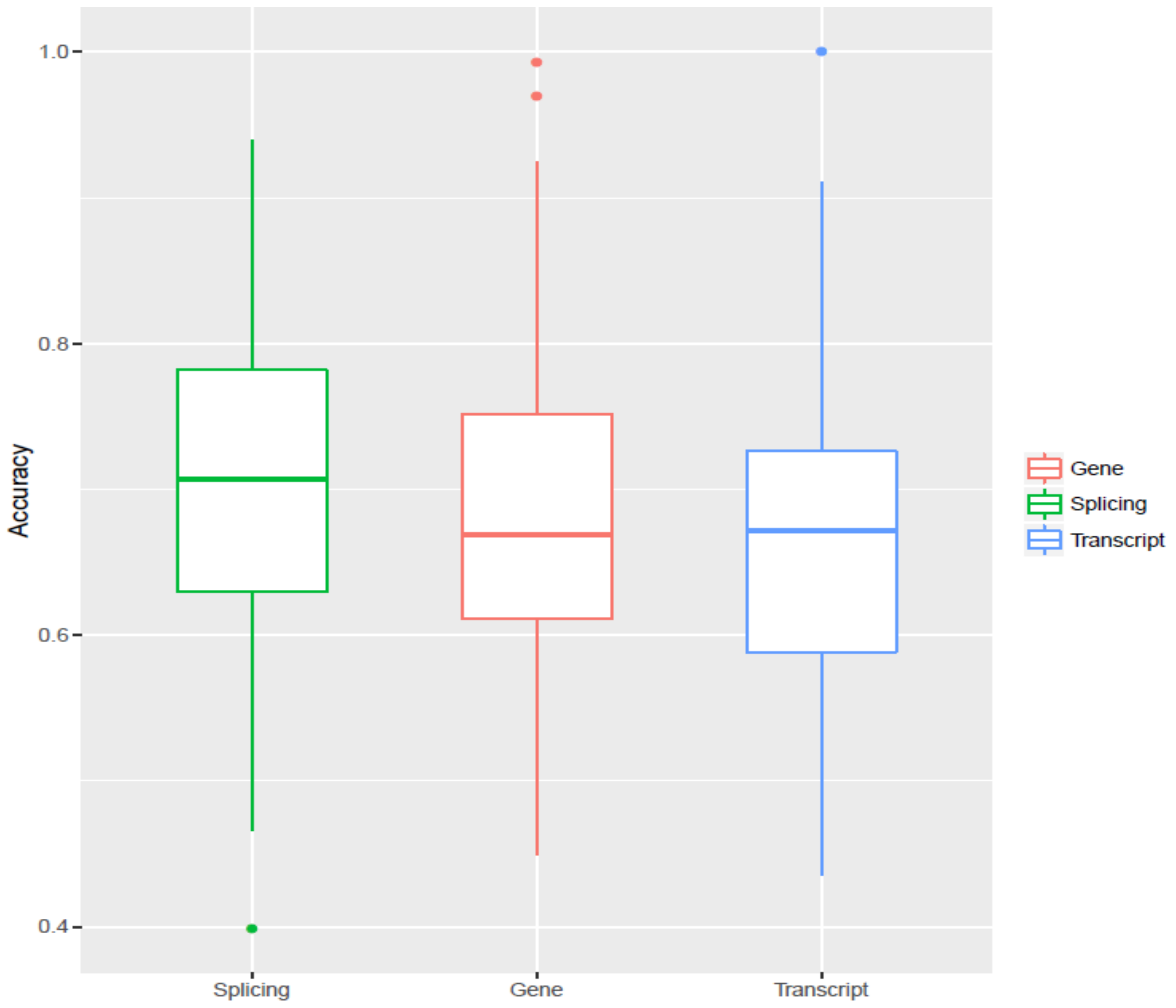

Figure S6: Age classification accuracy for three models. The green one is for splicing model, the red is for gene model, the blue is for transcript model.

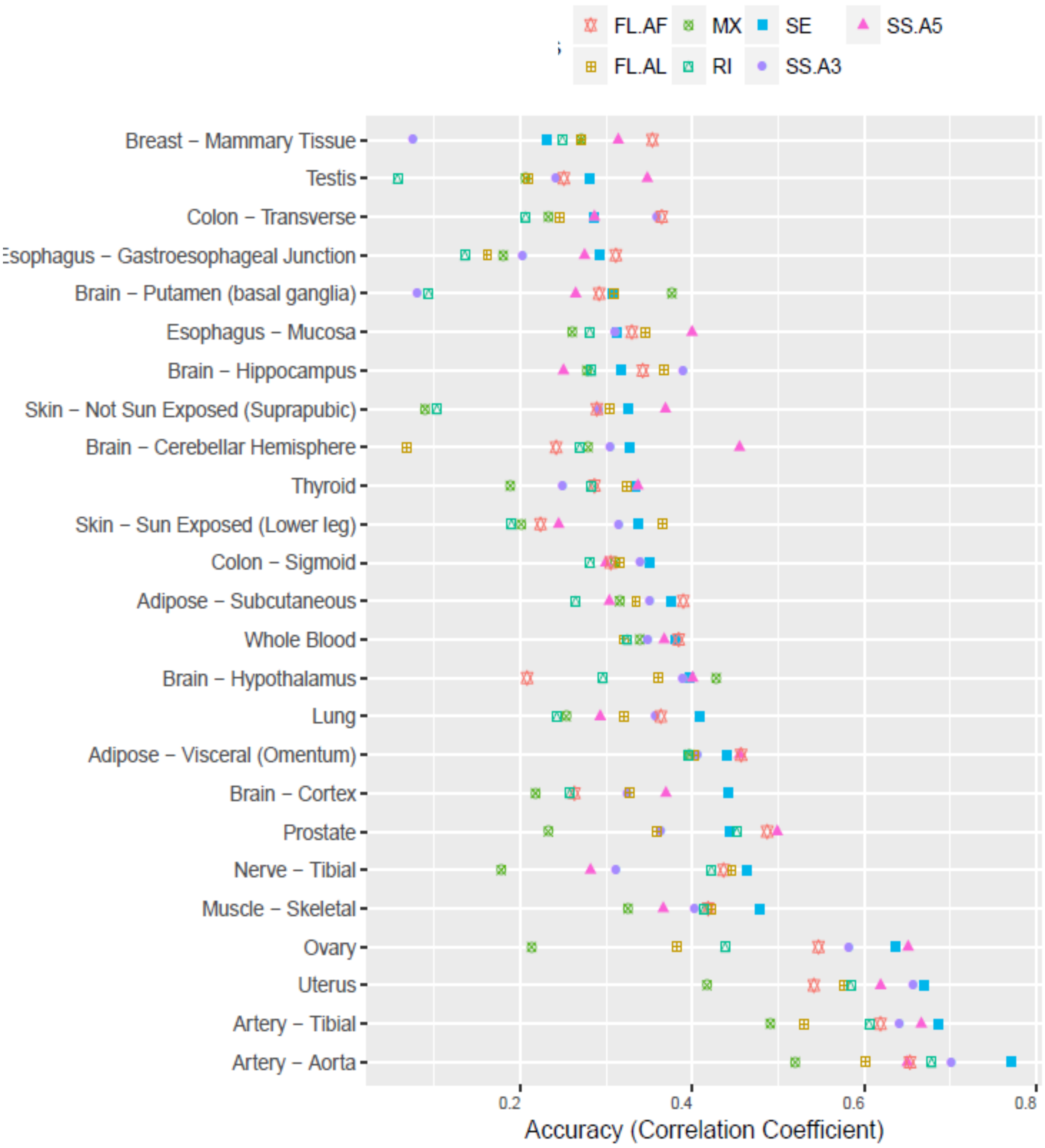

Figure S7: Accuracies of prediction of age using models based on 7 different types of splicing events across tissues. The symbols represent the 7 types of splicing events (as defined in Fig. 5). The accuracy is measured by Pearson correlation between predicted and true ages based on cross-validation.

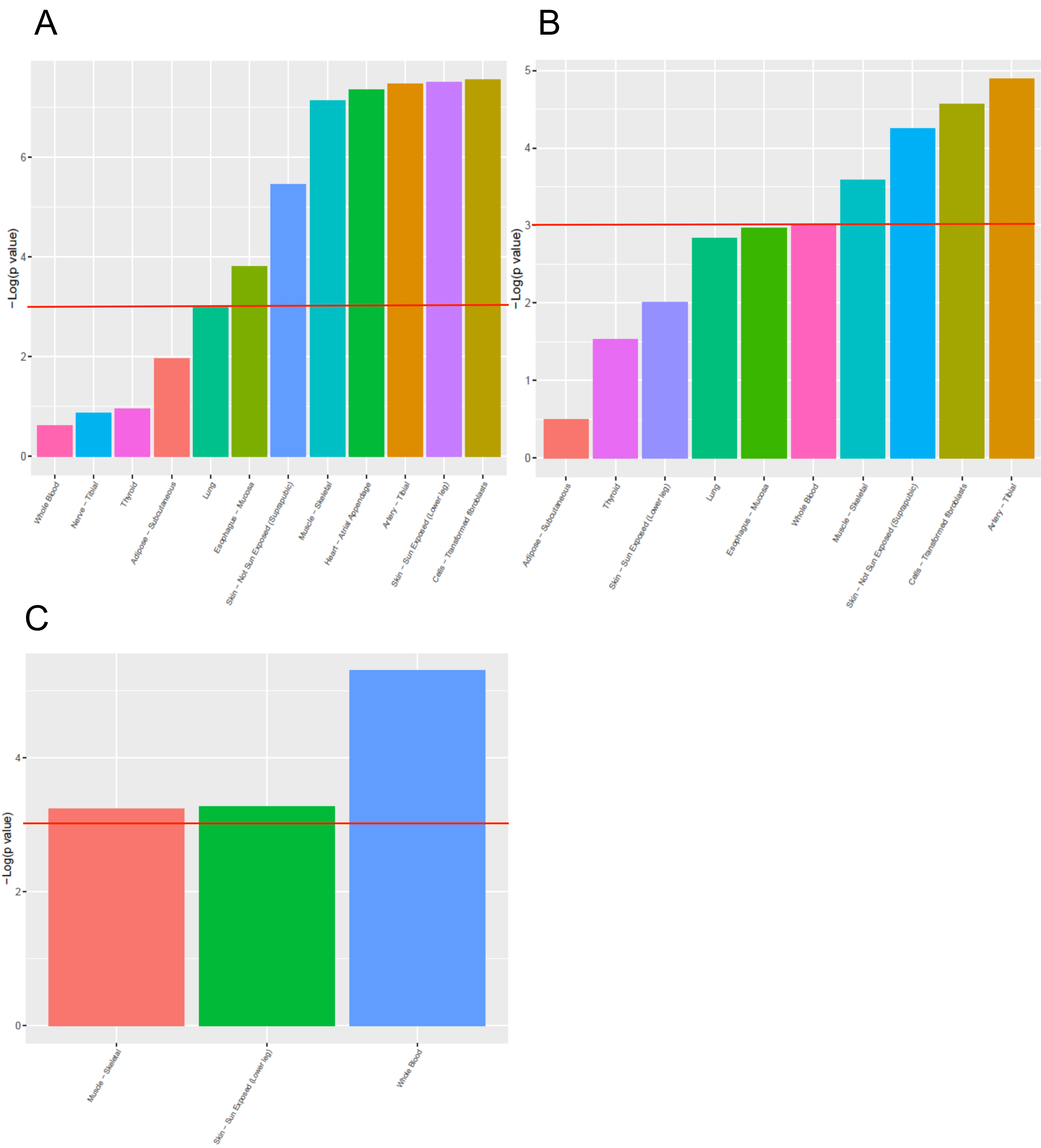

Figure S8: Additional contribution of splicing events to the explained variance of complex diseases: (A) Diabetes mellitus type II, (B) Chronic Respiratory Disease, (C) Heart Attack, in multiple tissues shown on x-axis. The y-axis denotes the significance (-log(p-value)) based on a log-likelihood ratio test. The red line indicates p-value = 0.05.

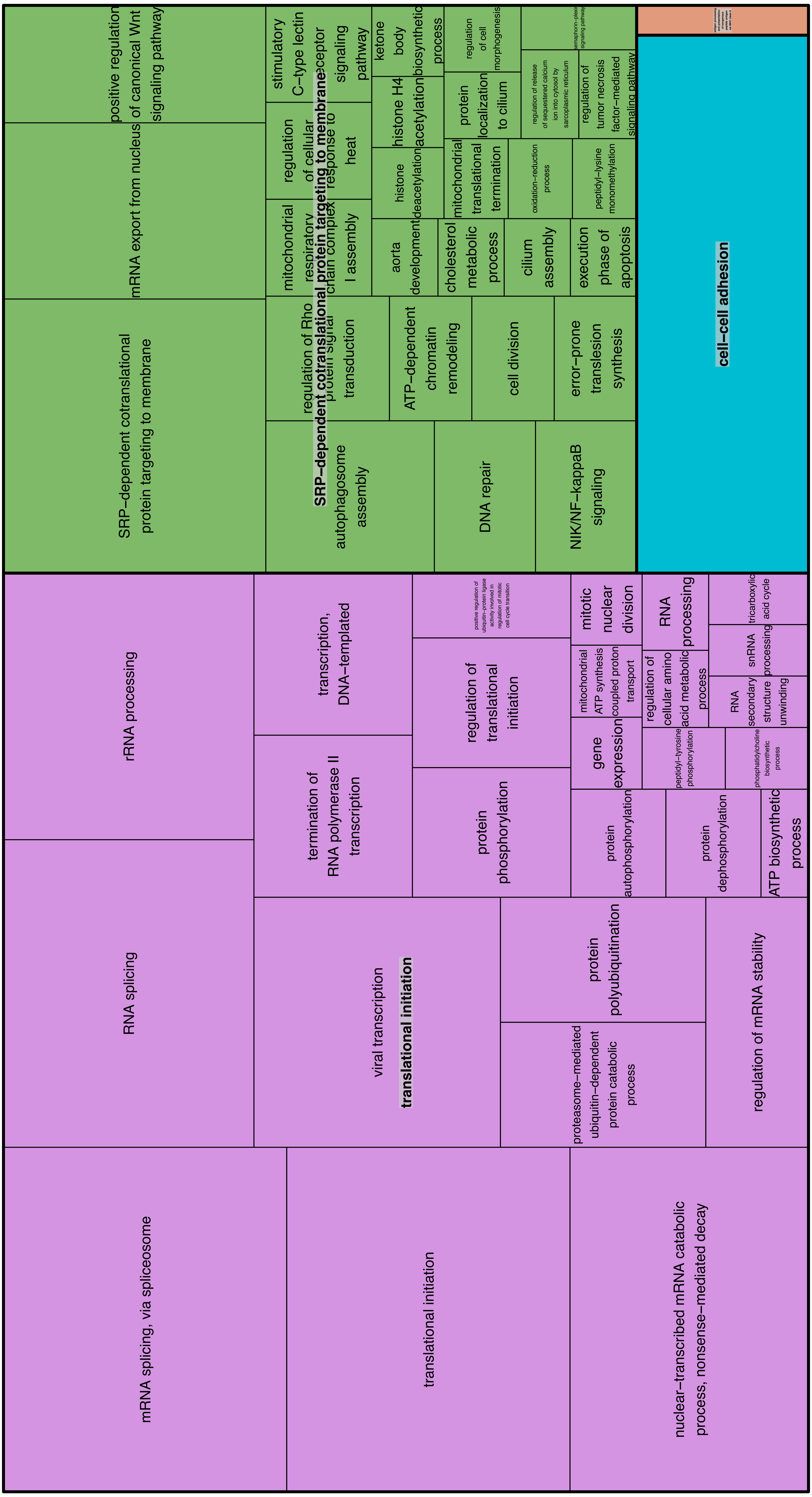

Figure S9: TreeMap view of GO terms enriched in at least one tissue. For each GO term, the number of tissues in which it is significant is used as the enrichment score for visualization.

Table S1: Number of significant age associated splicing events across tissues.

Tissue Types	Number of samples	SE (up)	SE (down)	MX (up)	MX (down)	SS.A5 (up)	SS.A5 (down)	SS.A3 (up)	SS.A3 (down)	FL.AF (up)	FL.AF (down)	FL.AL (up)	FL.AL (down)	RI (up)	RI (down)
Whole Blood	393	972	1207	0	1	41	44	81	71	11	12	39	54	2	0
Adipose - Subcutaneous	350	47	40	0	0	236	203	334	318	66	55	25	16	0	0
Muscle - Skeletal	430	56	34	7	1	20	21	49	29	14	11	80	69	20	4
Artery - Tibial	332	703	640	30	36	111	113	148	209	190	213	54	42	32	57
Artery - Coronary	133	3	6	0	0	0	3	0	0	0	0	0	0	1	0
Heart - Atrial Appendage	194	547	547	29	19	168	317	169	204	11	22	59	39	103	263
Adipose - Visceral (Omentum)	227	585	529	5	11	152	115	280	319	395	379	92	98	318	30
Ovary	97	0	0	23	13	29	35	0	4	3	2	160	149	0	0
Uterus	83	46	83	6	3	36	97	42	24	3	1	25	29	9	36
Vagina	96	0	0	30	27	0	0	0	0	0	0	1	1	81	303
Breast - Mammary Tissue	214	6	2	6	8	24	54	0	0	65	57	4	9	27	2
Skin - Not Sun Exposed (Suprapubic)	250	920	725	45	27	251	318	289	318	173	161	101	81	11	17
Minor Salivary Gland	57	0	0	0	0	0	0	0	0	0	0	0	0	0	0
Brain - Cortex	114	127	136	6	11	28	34	40	69	0	0	36	26	19	15
Adrenal Gland	145	5	3	0	0	3	2	0	0	0	1	7	4	0	0
Thyroid	323	10	6	21	25	211	455	280	424	1	0	53	58	60	93
Lung	320	154	244	0	1	320	255	181	142	43	45	71	80	0	0
Spleen	104	0	0	0	0	0	0	0	0	0	0	0	1	0	0
Pancreas	171	0	0	0	0	0	0	0	0	0	0	0	0	0	0
Esophagus - Muscularis	247	188	190	0	0	136	317	92	139	3	0	0	0	30	32
Esophagus - Mucosa	286	928	1161	32	42	278	220	284	231	824	788	196	203	52	87
Esophagus - Gastroesopha	153	61	48	3	1	40	20	49	24	162	157	21	32	105	221

geal Junction															
Stomach	193	115	62	29	18	123	153	291	328	2	8	191	157	198	45
Colon - Sigmoid	149	438	630	13	14	181	172	237	199	477	453	88	94	390	104
Small Intestine - Terminal Ileum	88	0	0	0	0	0	0	0	0	0	0	0	0	0	0
Colon - Transverse	196	69	111	3	2	119	137	312	271	39	46	26	29	497	41
Prostate	106	0	0	3	5	103	75	0	2	30	41	3	4	44	9
Testis	172	157	94	0	0	0	0	3	4	199	193	13	11	19	0
Skin - Sun Exposed (Lower leg)	357	1267	1025	0	0	365	216	291	216	4	5	153	150	41	60
Nerve - Tibial	304	935	675	14	18	130	300	237	468	12	14	135	126	21	15
Heart - Left Ventricle	218	26	50	2	2	22	20	266	195	2	4	39	38	93	16
Pituitary	103	0	0	0	0	0	0	2	0	0	0	1	1	0	0
Brain - Cerebellum	125	4	2	0	0	0	0	3	4	5	5	0	0	0	1
Cells - Transformed fibroblasts	284	106	97	2	5	26	55	17	38	142	142	21	22	32	21
Artery - Aorta	224	214	251	9	17	66	57	153	122	227	201	117	114	129	76
Cells - EBV-transformed lymphocytes	118	0	0	3	0	0	0	0	0	0	0	1	1	0	0
Liver	119	19	35	2	2	79	93	0	0	0	0	1	1	7	0
Brain - Hippocampus	94	43	12	1	5	0	1	20	12	0	0	2	5	83	6
Brain - Substantia nigra	63	0	3	9	7	16	36	18	33	79	56	0	0	6	13
Brain - Anterior cingulate cortex (BA24)	84	0	0	16	17	37	53	72	69	1	0	35	25	36	39
Brain - Frontal Cortex (BA9)	108	0	0	12	13	39	57	61	66	176	170	30	30	7	18
Brain -	105	0	0	1	6	43	58	44	57	25	20	34	24	44	31

Cerebellar Hemisphere															
Brain - Caudate (basal ganglia)	117	3	6	9	12	0	0	82	82	18	9	13	8	0	0
Brain - Nucleus accumbens (basal ganglia)	113	19	8	2	1	0	0	2	0	1	0	0	0	0	0
Brain - Putamen (basal ganglia)	97	0	0	5	7	0	0	21	29	1	0	0	0	0	0
Brain - Hypothalamus	96	2	4	6	8	0	0	17	17	20	5	0	0	0	0
Brain - Spinal cord (cervical c-1)	71	4	2	0	1	1	2	0	0	29	40	0	0	0	0
Brain - Amygdala	72	0	0	11	9	31	39	44	56	0	0	0	0	9	8

Table S2: Nineteen age-associated alternatively spliced genes shared by 9 tissues.

Gene name	Functions
PKD1	GO annotations related to this gene include protein kinase binding and protein domain specific binding
RNF10	GO annotations related to this gene include ubiquitin-protein transferase activity and transcription regulatory region DNA binding.
NFATC4	GO annotations related to this gene include transcription factor activity, sequence-specific DNA binding and transcription coactivator activity.
CLK4	GO annotations related to this gene include transferase activity, transferring phosphorus-containing groups and protein tyrosine kinase activity.
HDLBP	GO annotations related to this gene include nucleic acid binding and RNA binding.
ZMYM6	GO annotations related to this gene include nucleic acid binding
LINC00963	long non-coding RNA
TCOF1	GO annotations related to this gene include poly(A) RNA binding and transporter activity.
ITGA7	GO annotations related to this gene include protein heterodimerization activity and cell adhesion molecule binding.
CNTROB	GO annotations related to this gene include protein domain specific binding
WDR73	May play a role in the regulation of microtubule organization and dynamics
VEZT	GO annotations related to this gene include myosin binding.
ARHGEF1	GO annotations related to this gene include poly(A) RNA binding and Rho guanyl-nucleotide exchange factor activity.
ANAPC5	GO annotations related to this gene include protein phosphatase binding.
LONP1	GO annotations related to this gene include sequence-specific DNA binding and serine-type endopeptidase activity
STARD3	GO annotations related to this gene include lipid binding and cholesterol transporter activity
TPM1	GO annotations related to this gene include actin binding and cytoskeletal protein binding
UBA5	GO annotations related to this gene include UFM1 activating enzyme activity
MFF	GO annotations related to this gene include protein homodimerization activity.

Table S3: Top 15 biological function terms based on number of tissues affected and enrichment (Fold Change).

Top Functional terms based on number of Tissues affected	Top Functional terms based on Fold Change
Ribonucleoprotein	large ribosomal subunit rRNA binding
cadherin binding involved in cell-cell adhesion	Peroxisome biogenesis
mRNA processing	protein import into peroxisome matrix
mRNA splicing, via spliceosome	RS domain binding
translational initiation	peptidyl-lysine monomethylation
cell-cell adhesion	execution phase of apoptosis
Transit peptide	mitochondrial proton-transporting ATP synthase complex, coupling factor F(o)
ATP-binding	activating transcription factor binding
Nucleotide-binding	histone H4 acetylation
Mitochondrion	hsa04970:Salivary secretion
translation	regulation of release of sequestered calcium ion into cytosol by sarcoplasmic reticulum
transit peptide: Mitochondrion	Viral nucleoprotein
nuclear-transcribed mRNA catabolic process, nonsense-mediated decay	translation initiation factor activity
ribosome	error-prone translesion synthesis
Metal-binding	protein import into nucleus, docking

Table S4: Potential splicing factor drivers in skin fibroblast tissue.

Potential Driver	Location
SRSF7	I1/I4
SNRPA	I1
U2AF2	E2
HNRNPC	E2
PTBP1	E2
SRSF2	E2
TIA1	E2
TIAL1	E2
RALY	E2

Table S5: Number of tested events across 48 tissues.

	SE	MX	SS.A5	SS.A3	FL.AF	FL.AL	RI
Total Number of Events	35877	4626	13787	14387	68176	17552	5509
Whole Blood	21499	1814	8370	9199	29162	8091	4279
Adipose - Subcutaneous	23679	2049	9320	9843	32910	9208	4480
Muscle - Skeletal	23073	2007	9022	9553	32965	8971	4328
Artery - Tibial	23162	2011	9025	9560	32402	9044	4360
Artery - Coronary	21118	1664	8202	8877	26210	7518	4220
Heart - Atrial Appendage	22286	1824	8621	9325	29712	8375	4323
Adipose - Visceral (Omentum)	22923	1927	8927	9611	30636	8678	4450
Ovary	19880	1442	7867	8440	23610	6973	4113
Uterus	19601	1378	7694	8327	22607	6605	4078
Vagina	20461	1462	8083	8698	24301	6977	4223
Breast - Mammary Tissue	23020	1915	9029	9671	30945	8716	4475
Skin - Not Sun Exposed (Suprapubic)	22342	1830	8791	9495	29592	8394	4438
Minor Salivary Gland	18024	1062	6974	7693	18381	5268	3870
Brain - Cortex	19224	1419	7623	8288	23099	6743	4096
Adrenal Gland	21553	1699	8530	9154	27987	7919	4284
Thyroid	23333	2014	9207	9852	31971	9024	4547
Lung	24414	2125	9496	10253	33714	9386	4632
Spleen	18699	1358	7498	8210	21336	6475	4153
Pancreas	20358	1558	8073	8734	25505	7284	4226
Esophagus - Muscularis	22290	1868	8764	9393	30012	8470	4369
Esophagus - Mucosa	23495	2010	9297	9834	32795	8862	4512
Esophagus - Gastroesophageal Junction	20795	1597	8159	8838	26317	7575	4229
Stomach	22078	1760	8614	9392	28532	7993	4406
Colon - Sigmoid	21035	1609	8203	8949	26175	7555	4233
Small Intestine - Terminal Ileum	20009	1433	7865	8640	23292	6656	4264
Colon - Transverse	22556	1836	8832	9574	29592	8275	4476
Prostate	19788	1413	7809	8531	23197	6845	4238
Testis	26176	2326	10199	10756	35594	10418	4736
Skin - Sun Exposed (Lower leg)	23192	1974	9181	9854	31913	9008	4515
Nerve - Tibial	22831	1953	8987	9613	30718	8810	4455
Heart - Left Ventricle	21299	1727	8367	8984	27955	7965	4188
Pituitary	19805	1435	7833	8524	23339	6898	4212
Brain - Cerebellum	19422	1450	7745	8402	23815	6804	4135
Cells - Transformed fibroblasts	24123	2239	9443	9708	36058	9575	4196
Artery - Aorta	22125	1846	8695	9319	29445	8409	4345
Cells - EBV-transformed lymphocytes	21900	1898	8685	9021	29915	8065	4050
Liver	18280	1265	7186	7963	20563	6177	4018
Brain - Hippocampus	18070	1205	7089	7805	20016	5919	3896
Brain - Substantia nigra	16362	976	6298	7049	15912	4700	3614
Brain - Anterior cingulate cortex (BA24)	18160	1209	7112	7800	20241	5878	3893
Brain - Frontal Cortex (BA9)	19387	1411	7601	8290	23131	6669	4010

**Brain - Cerebellar Hemisphere**	19362	1407	7626	8310	23118	6566	4045
**Brain - Caudate (basal ganglia)**	19488	1413	7639	8339	23329	6826	4071
**Brain - Nucleus accumbens (basal ganglia)**	19693	1433	7678	8378	23562	6760	4097
**Brain - Putamen (basal ganglia)**	18125	1228	7070	7786	20344	5991	3908
**Brain - Hypothalamus**	19086	1318	7449	8196	21790	6420	4021
**Brain - Spinal cord (cervical c-1)**	17546	1125	6795	7487	18105	5293	3756
**Brain - Amygdala**	16970	1062	6575	7261	17426	5125	3723

Supplementary Note 1

We ascertained that the number of significant events detected was not correlated with the number of samples across tissues; Pearson correlation between sample size and fraction of events found to be 0.06 (p-value = 0.8). We also assessed, for each event type, the potential bias between up-regulated and down-regulated events using paired Wilcoxon test, and found most types of splicing events to be relatively balanced. There was a modest bias in alternative 5' usage (toward down-regulated events; p-value = 0.047) and alternative first exons events (toward longer isoforms; p-value = 0.03).

Supplementary Note 2

PEER factors are expected to capture a variety of known and hidden confounders such as batch effects and global population variance. We explicitly remove the PEER factors that are correlated with age to minimize false negatives, because such PEER factors will explicitly occlude the associations between specific splicing events and age. Since some of the removed PEER factors are also correlated with race or ethnicity, we may falsely detect splicing events associated with race/ethnicity instead of age. Now we have tested whether our results are robust to human ancestry or not.

Recall that in our default approach we removed all PEER factors associated with age, with no consideration to ancestry. In Skin (sun exposed) tissue, for instance, we found that on average across all 23192 exon Skipping events, (1) Of the removed factors, almost none (8e-3%) are correlated with ethnicity and 2.4% are correlated with race. (2) even if we include PEER factors correlated with ethnicity (and age), all detected events were still deemed insignificant, and (3) if we include PEER factors correlated with race (and age), only 0.05% of detected events failed to qualify. We have done this assessment for all tissues and found that (1) Of the removed factors, on average 4.8% are correlated with ethnicity and on average 8.2% are correlated with race. (2) If we include PEER factors correlated with ethnicity, on average only 0.3% of detected events would be deemed insignificant, and (3) if we include PEER factors correlated with race, around 0.35% of detected events would be deemed insignificant in 45 tissues. However, we observed significant drop in detectability in three tissues (Blood, Lung and Colon – Sigmoid), prompting us to perform yet another robustness analysis.

As a potential cause for the drop in detectability in 3 tissues, consider the following. Since age and race can indeed be correlated due to population sampling bias, retaining race-associated PEER factor (thus indirectly retaining age-associated factors), we inadvertently control for age, which can lead to substantial false negatives. Thus instead of including PEER factors correlated with both age and race, we evaluated the model by directly including race as a confounding covariate (ethnicity information is too sparse to perform this analysis). We found that on average only 3% of detected events are deemed insignificant, and importantly, the three tissues showing substantial difference above do not show substantial differences any more (only ~5%).

Thus, overall, the possible false positive due to exclusion of PEER factors that might be associated with race/ethnicity is not substantial and our model should be robust to human ancestry.
